# Theoretical Insight into the Biodegradation of Solitary Oil Microdroplets Moving through a Water Column

**DOI:** 10.3390/bioengineering5010015

**Published:** 2018-02-12

**Authors:** George E. Kapellos, Christakis A. Paraskeva, Nicolas Kalogerakis, Patrick S. Doyle

**Affiliations:** 1 Department of Chemical Engineering, Massachusetts Institute of Technology, Cambridge, MA 02139, USA; pdoyle@mit.edu; 2 School of Environmental Engineering, Technical University of Crete, 73100 Chania, Greece; nicolas.kalogerakis@enveng.tuc.gr; 3 Department of Chemical Engineering, University of Patras, 26504 Rion Achaia, Greece; takisp@chemeng.upatras.gr

**Keywords:** biofilm, crude oil, modeling, oil spill, droplet cloud, droplet dissolution, droplet biodegradation, Sherwood number, mass transfer, compound droplet model

## Abstract

In the aftermath of oil spills in the sea, clouds of droplets drift into the seawater column and are carried away by sea currents. The fate of the drifting droplets is determined by natural attenuation processes, mainly dissolution into the seawater and biodegradation by oil-degrading microbial communities. Specifically, microbes have developed three fundamental strategies for accessing and assimilating oily substrates. Depending on their affinity for the oily phase and ability to proliferate in multicellular structures, microbes might either attach to the oil surface and directly uptake compounds from the oily phase, or grow suspended in the aqueous phase consuming solubilized oil, or form three-dimensional biofilms over the oil–water interface. In this work, a compound particle model that accounts for all three microbial strategies is developed for the biodegradation of solitary oil microdroplets moving through a water column. Under a set of educated hypotheses, the hydrodynamics and solute transport problems are amenable to analytical solutions and a closed-form correlation is established for the overall dissolution rate as a function of the Thiele modulus, the Biot number and other key parameters. Moreover, two coupled ordinary differential equations are formulated for the evolution of the particle size and used to investigate the impact of the dissolution and biodegradation processes on the droplet shrinking rate.

## 1. Introduction

After a natural or accidental release of crude oil in the sea, part of the oil ends up in the form of droplets moving through the seawater column. The droplets may be created either at the sea surface during the breakup of an oil slick (i.e., floating oil layer) by sea waves [1,2], or at the seafloor during the atomization of live crude oil (i.e., gas/oil mixture) extruding at sufficiently high speed from a natural crack or a broken wellhead [3,4,5]. The latter case occurred, for example, after the blowout of the Deepwater Horizon rig in the Gulf of Mexico where the addition of the chemical dispersant Corexit in the leaking crude oil resulted in clouds of droplets travelling underwater along with sea currents [6,7]. At present, there are no practical means for the collection or in situ treatment of oil droplets in vast bodies of marine waters and, inevitably, their removal relies solely on natural attenuation processes, notably on dissolution and biodegradation. Specifically, it is anticipated that in the long run, most of the released oil in the sea is consumed by autochthonous oil-degrading microorganisms (bacteria, fungi, yeasts) that have developed appropriate machinery for accessing and assimilating oily substrates [8,9,10]. In this way, crude oil enters as a nutrient into the marine food chain. In spite of this long-term bright side, large amounts of dispersed oil droplets in the seawater column disturb the established ecosystem dynamics and pose an imminent risk of toxic effects from various crude oil components to many marine species (invertebrates, fishes, mammals, etc.) [11,12,13,14]. In particular, small oil droplets might be more toxic than crude oil itself, if consumed by fish and marine mammals [14]. It is therefore imperative to understand and quantify the physical and biological mechanisms that rule the fate of dispersed oil droplets in marine waters and, upon that knowledge, build technologies that will enable the mitigation of pertinent adverse effects. 

Once the droplets entrain to the seawater column, the most critical quantity to assess is the droplet *retention time* in the underwater body until complete dissolution, degradation or relocation to the sea surface or seafloor. The retention time depends strongly on the direction of droplet motion and the rate of droplet shrinking. Dispersed droplets might be rising, settling, or drifting along sea currents. The detailed motion of the droplets depends on a number of factors, including the physical properties of the oil–water system (density, viscosity, interfacial tension), the temperature profile, the droplet size, the composition of the oil surface, the presence of marine snow and snot, and the flow direction and strength of underwater currents [15]. Under the action of buoyancy, large drops (>2 mm) and oil blobs rise towards the sea surface where they (re)coalesce with the oil slick. On the other hand, microdroplets with a size in the range of 10–100 µm have a lower rise velocity and higher probability of being carried away by underwater currents. Adsorption of chemical dispersants or naturally-occurring colloids and surfactants to the droplet surface hinders the tangential mobility of the oil–water interface, reduces the recirculating flow within the droplet, retards the overall motion of the droplet, and prevents droplet–droplet coalescence [16]. Interaction of the drifting droplets with settling marine snow (i.e., plankton and suspended microbial flocs) may lead to the formation of complex aggregates that tend to settle down on the seafloor [17,18], and stimulate chemotactic responses of other oil-degrading microbial species residing in sediments [19,20]. The probability of collision between marine snow and oil droplets depends on the concentration and size distribution of the two particulate populations. A higher concentration of larger particles creates a higher probability of aggregation and sedimentation [21]. In general, the combined effects of smaller size and interfacial contamination result in a higher probability of microdroplets forming stable droplet clouds with significant retention time in the seawater column. 

The droplet shrinking rate is determined by the dissolution and biodegradation processes. The dissolution rate depends on the solubility of oil in water, the diffusivity of oil in water, and the velocity of the surrounding fluid relative to the droplet [16,22]. The solubility of most oil compounds is rather low, but may be enhanced by the action of surfactant micelles [23,24,25,26,27]. The biodegradation rate depends on the microbial strategy for oil uptake, the concentration of microbes, the intrinsic kinetics for oil consumption, the physical conditions (temperature, pressure, pH, salinity) and the availability of electron acceptors and mineral nutrients [8,9,10,28,29,30,31].

Three major microbial strategies have been identified for accessing and assimilating oily substrates; an outline is given here and a more detailed discussion is available in [31]. In a first strategy, microbes *firmly adhere* to the oil–water interface and sip oil compounds directly from the oily phase. This approach has been observed in pure cultures of super-hydrophobic, Gram-positive microbes, like *Mycobacterium* and *Rhodococcus* species. In a second strategy, microbes grow *suspended* in the bulk aqueous phase and uptake-dissolved and micellar oil compounds. This strategy has been observed, for example, in pure cultures of Gram-negative microbes, mainly of *Pseudomonas* species, that have a hydrophilic cell surface and produce biosurfactants of low molecular weight (e.g., rhamnolipids). In a third strategy, individual or clustered microbes adhere to the oil surface and actively *form biofilms* by secreting excessive amounts of biopolymers with high molecular weight. The biopolymers, mainly polysaccharides and proteins, do not dissolve into the bulk aqueous phase, but instead accumulate in the extracellular space and spontaneously assemble to form a three-dimensional matrix enmeshing the cells. The biofilm growth mode over oily substrates has been reported for several pure cultures and mixed microbial consortia. Current theoretical models for the fate of oil droplets in marine waters account only for the direct uptake strategy [32,33,34], neglecting any effects of biodegradation in the bulk aqueous phase or the formation of biofilm over the droplet and bioreaction therein. 

**Figure 1:**
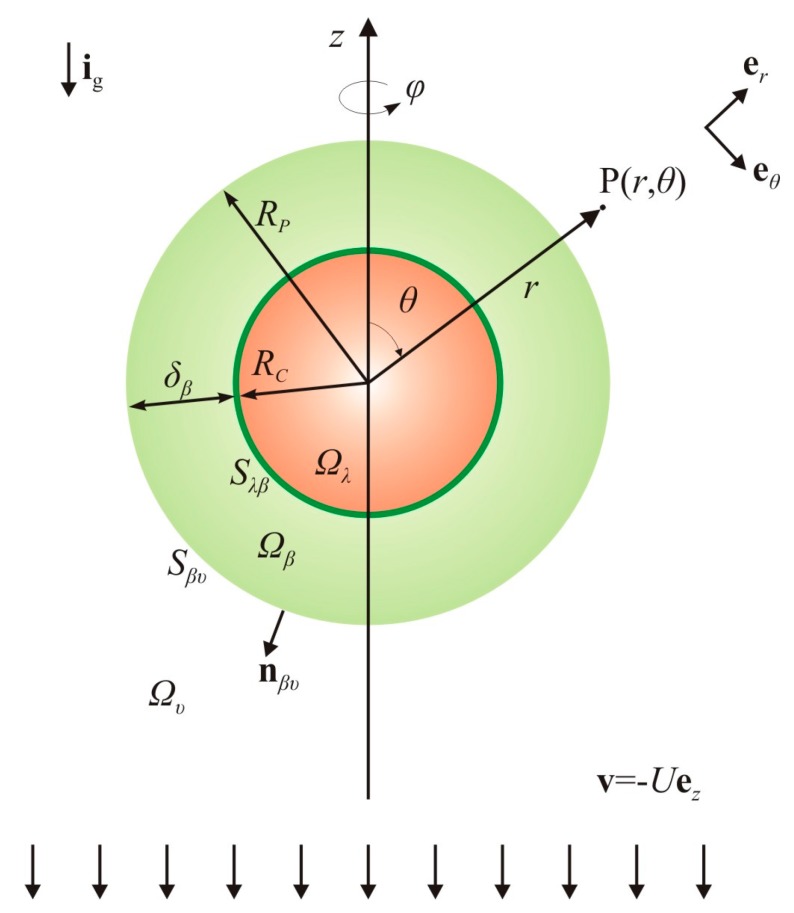
Geometry and coordinate system for the compound particle model (description in the text).

In this work, a compound particle model (CPM) is developed for the biodegradation of solitary oil microdroplets moving through a water column. The compound particle is of the core-shell type and consists of an oily core that is successively surrounded by a bioreactive skin of negligible thickness and another bioreactive shell of finite thickness (Figure 1). The bioreactive skin represents a thin layer of microbes that uptake oil directly from the oily phase, whereas the bioreactive shell represents a distinct biofilm phase. In line with the abovementioned microbial strategies of oil uptake, the model accounts for all three modes of biodegradation: direct interfacial uptake, bioreaction in the bulk aqueous phase, and bioreaction in a biofilm formed around the droplet. A set of simplifying hypotheses is introduced so as to make the mathematical analysis tractable, and the governing equations solvable by analytical methods. The most important hypotheses are that the compound particle is considered to move as a non-deforming rigid sphere, the flow of the aqueous phase is dominated by viscous stresses, and the transport of dissolved oil in the biofilm phase is dominated by diffusion, whereas in the bulk aqueous phase it is dominated by advection. The analysis of the local mass balances results in a closed-form expression for the overall dissolution rate as a function of the Biot number, the Thiele modulus, the thickness of the biofilm, and the diffusivity and solubility ratios. Furthermore, from the overall mass balances, two coupled ordinary differential equations are established for the evolution of the particle size.

## 2. Model Formulation

With reference to Figure 1, the process under consideration is the transport and reaction of dissolved oil, denoted as the *A* solute, from the oil droplet (Ω_λ_) to the surrounding biofilm (Ω_β_) and aqueous (Ω_υ_) phases. The thick line at the oil–biofilm interface (S_λβ_) represents a thin layer of microbes that uptake oil compounds directly from the oily phase. The first step in the theoretical analysis is to determine the oil dissolution rate at the droplet surface, based on an appropriate formulation of the *local* mass balances (Section 2.3). The second step is to determine the droplet shrinking rate using the *overall* mass balances (Section 2.4). Before proceeding with the mathematical analysis, certain key considerations on modeling the different biodegradation modes (Section 2.1) and a set of basic hypotheses (Section 2.2) are set forth.

### 2.1. Considerations on Modeling the Three Major Biodegradation Modes

A few remarks are in order with regard to the theoretical modeling of each one of the three basic modes of biodegradation; that is, direct interfacial uptake, bioreaction in the bulk aqueous phase, and bioreaction in a biofilm formed around the droplet. 

The first type of oil-degrading microbes is the *flatlanders*; that is superhydrophobic microbes able to firmly adhere to the oil surface and directly uptake organic compounds from the oily phase. Here, it is considered that the oil surface (S_λβ_) is fully and uniformly covered by flatlanders. Partial coverage is expected to lead to more complex phenomena of fluid dynamics and solute transport and, thus, deserves to be investigated separately. The layer of flatlanders is usually found embedded in the oil side of the oil–water interface [35] and can be viewed as a *bioreactive skin* (interphase) of negligible thickness (~ a few µm) on the droplet scale of observation (~100 µm). As the microbes have direct access to the oily substrate, the oil consumption rate is considered to be limited only by the intrinsic microbial kinetics. Under these conditions, this mode of biodegradation is essentially decoupled from the dissolution of oil to the surrounding phases. The physical presence of microbes on the droplet surface and the process of interfacial reaction are assumed to affect only implicitly the dissolution of oil; that is, by (possibly) changing the value of oil solubility. 

The second type of oil-degrading microbes is the *drifters*; that is, hydrophilic microbes that remain suspended in the bulk aqueous phase (Ω_υ_) and consume solubilized (molecular or micellar) oil. For this biodegradation mode, it is considered that the concentration of microbes is constant throughout the aqueous phase and the oil consumption rate follows first-order kinetics. In addition, solute A represents both molecular and micellar oil and, thus, the action of surfactants is taken into account only implicitly by modifying the apparent solubility of oil in the aqueous phase.

The third type of oil-degrading microbes is the *biofilm* formers; that is, microbes able to actively construct three-dimensional biofilm communities over the oil surface. The thickness of the biofilm might be appreciable and, thus, the biofilm is viewed as a distinct phase on the droplet scale of observation. Supplementary hypotheses for this mode include a uniform biofilm thickness, constant concentration of active microbes within the biofilm, and first order kinetics for the oil consumption rate. Interstitial flow is neglected and solute transport within the biofilm is dominated by diffusion.

With regard to the microbial proliferation rate, it is customary to assume a linear dependence on the concentration of active cells, that is

(1) $${\tilde{r}}_{C,\alpha }={\tilde{\mu }}_{C,\alpha }{\tilde{B}}_{\alpha },$$

where α denotes the physical domain in which the microbes grow and takes the values α=β, υ, λβ. All of the primary symbols are defined in the nomenclature. The tilde (~) over a variable or parameter denotes a dimensional quantity, whereas the lack of it denotes a dimless quantity. The term dimensionless is abbreviated to *dimless* throughout the paper. The specific growth rate ${\tilde{\mu }}_{C,\alpha }$ is usually considered to follow Monod kinetics.

(2) $${\tilde{\mu }}_{C,\alpha }=\frac{{\tilde{\mu }}_{m,\alpha }{\tilde{c}}_{A\alpha }}{{\tilde{K}}_{S,\alpha }+{\tilde{c}}_{A\alpha }}.$$

Any possible effects of lag phase, cell maintenance, limitation by electron acceptors, nitrogen and phosphorous sources, substrate cometabolism or inhibition are neglected. Of particular interest are the limiting forms for the specific growth rate under *sufficiently high or low* concentration.

(3) $${\tilde{\mu }}_{C,\alpha }≅\{\begin{array}{cc} {\tilde{\mu }}_{m,\alpha } & if\;{\tilde{c}}_{A\alpha }\gg {\tilde{K}}_{S,\alpha }\\ \frac{{\tilde{\mu }}_{m,\alpha }}{{\tilde{K}}_{S,\alpha }}{\tilde{c}}_{A\alpha } & if\;{\tilde{c}}_{A\alpha }\ll {\tilde{K}}_{S,\alpha } \end{array}.$$

The zeroth-order kinetics is expected to be applicable in the interfacial uptake mode because the microbial cells have access to the pure oily phase. On the other hand, the first-order kinetics is expected to be applicable in the suspended and biofilm growth modes because of the low solubility of oil compounds in aqueous phases. In all cases, the oil consumption rate is considered to be proportional to the cell proliferation rate. Therefore, the volumetric consumption rate of dissolved oil in a bulk phase, is given by

(4) $${\tilde{r}}_{A,\alpha }=-\frac{{\tilde{r}}_{C,\alpha }}{Y_{C/A,\alpha }}=-{\tilde{k}}_{1\alpha }{\tilde{c}}_{A\alpha },\;with\;{\tilde{k}}_{1\alpha }=\frac{{\tilde{\mu }}_{m,\alpha }{\tilde{B}}_{\alpha }}{{\tilde{K}}_{S,\alpha }Y_{C/A,\alpha }},$$

for *α* = *β*, *υ*; and the surficial consumption rate on the droplet surface, is given by

(5) $${\tilde{r}}_{A,\lambda \beta }=-\frac{{\tilde{\mu }}_{m,\lambda \beta }}{Y_{C/A,\lambda \beta }}{\tilde{B}}_{\lambda \beta }.$$

Here, ${\tilde{B}}_{\alpha }$ and ${\tilde{B}}_{\lambda \beta }$ is the volumetric and surface concentration of cells, respectively. The minus sign denotes consumption rates. In this work, the cell concentration is treated as a constant for all three biodegradation modes. 

### 2.2. Basic Hypotheses for the Hydrodynamics and Mass Transport

In addition to the previous considerations for the biodegradation process, a set of hypotheses is introduced for the flow and mass transport processes so as to simplify the mathematical description as much as possible while retaining the most important mechanisms. First, the external flow in the unbounded aqueous phase is dominated by viscous stresses and, thus, characterized by a low Reynolds number (Reυ=R˜PU˜ρ˜υ/μ˜υ≪1). Second, the internal recirculating flow and the deformation of the particle are considered to be negligible. In all cases, the adsorption of biopolymers and microbial cells to the oil–water interface is expected to hinder the interfacial mobility and, consequently, diminish the internal flow in the oily phase. On the basis of a combination of small particle size, slow velocity and rigid-like interface, it is expected that interfacial tension dominates over viscous and gravitational forces that tend to deform the particle and the system is, thus, characterized by low capillary and Bond numbers ($\mathrm{Ca}={\tilde{\mu }}_{\upsilon }\tilde{U}/{\tilde{\gamma }}_{\beta \upsilon }\ll 1$, $\mathrm{Bo}={\tilde{R}}_{P}^{2}\Delta \tilde{\rho }\tilde{g}/{\tilde{\gamma }}_{\beta \upsilon }\ll 1$; where $\Delta \tilde{\rho }=\left|{\tilde{\rho }}_{\upsilon }-{\tilde{\rho }}_{p}\right|$ is the excess density and ${\tilde{\gamma }}_{\beta \upsilon }$ is the interfacial tension at the particle surface) [36]. Therefore, the particle, either simple or compound, is considered to move as a rigid sphere. Third, the transport of dissolved oil in the biofilm phase is dominated by diffusion and, thus, characterized by a low Péclet number (${\mathrm{Pe}}_{\beta }={\tilde{R}}_{P}{\tilde{U}}_{\beta }/{\tilde{D}}_{A\beta }\ll 1$). On the other hand, mass transport in the bulk aqueous phase is considered to be dominated by advection and characterized by a high Péclet number (${\mathrm{Pe}}_{\upsilon }={\tilde{R}}_{P}\tilde{U}/{\tilde{D}}_{A\upsilon }\gg 1$). In both phases, solute diffusion is considered to obey Fick’s constitutive law. Fourth, the oily phase is treated as a single compound and mass transfer therein is not taken into account (e.g., the solute *A* represents the total petroleum hydrocarbon in the case of crude oil). Finally, the quasi-steady state hypothesis is adopted for the analysis of the flow and mass transport problems at the local level (Section 2.3). Besides a high Péclet and a low Reynolds number, this assumption also requires a low droplet shrinking rate as compared to the characteristic velocity of the external flow. Thereafter, the evolution of the particle size is treated as a sequence of steady states in Section 2.4.

### 2.3. Overall Dissolution Rate: Analysis of the Local Mass Balances

Under the detailed set of considerations and hypotheses given in the previous subsections, mass transport is described in the context of the CPM by the following equations

(6a) $${\tilde{c}}_{A\beta }={\tilde{c}}_{A,\lambda /\beta },\;\mathrm{at}\;\tilde{r}={\tilde{R}}_{C},$$

(6b) $$0={\tilde{D}}_{A\beta }{\tilde{\nabla }}^{2}{\tilde{c}}_{A\beta }-{\tilde{k}}_{1\beta }{\tilde{c}}_{A\beta },\;\mathrm{in}\;\mathrm{the}\;\beta -\mathrm{phase},$$

(6c) $${\tilde{J}}_{A\beta }\cdot n_{\beta \upsilon }={\tilde{J}}_{A\upsilon }\cdot n_{\beta \upsilon },\;\mathrm{at}\;\tilde{r}={\tilde{R}}_{P},$$

(6d) $$H_{A,\upsilon /\beta }{\tilde{c}}_{A\beta }={\tilde{c}}_{A\upsilon },\;\mathrm{at}\;\tilde{r}={\tilde{R}}_{P},$$

(6e) $${\tilde{v}}_{\upsilon }\cdot \tilde{\nabla }{\tilde{c}}_{A\upsilon }={\tilde{D}}_{A\upsilon }{\tilde{\nabla }}^{2}{\tilde{c}}_{A\upsilon }-{\tilde{k}}_{1\upsilon }{\tilde{c}}_{A\upsilon },\;\mathrm{in}\;\mathrm{the}\;\upsilon -\mathrm{phase},$$

(6f) $${\tilde{c}}_{A\upsilon }=0,\;\mathrm{at}\;\tilde{r}\to \infty .$$

It is possible to further reduce the complexity of the above set of governing equations by introducing two *educated hypotheses*. First, the tangential diffusion in the spherical shell is neglected. Strictly, this hypothesis holds for a thin shell (${\tilde{\delta }}_{\beta }\ll {\tilde{R}}_{P}$) or fast reaction (${\mathrm{Da}}_{\beta }\gg 1$). Thus, Equation (6b) becomes

(7) $$0=\frac{{\tilde{D}}_{A\beta }}{{\tilde{r}}^{2}}\frac{d}{d\tilde{r}}\left({\tilde{r}}^{2}\frac{d{\tilde{c}}_{A\beta }}{d\tilde{r}}\right)-{\tilde{k}}_{1\beta }{\tilde{c}}_{A\beta },\;\mathrm{in}\;\mathrm{the}\;\beta -\mathrm{phase}.$$

Second, the continuity of the mass flux at the υβ-interface is imposed in an *average sense* by demanding equality of the surface-averaged fluxes, instead of equality of the local fluxes. Therefore, Equation (6c) is expressed as follows

(8a) $$\overset{}{\underset{S_{\beta \upsilon }}{\int }}{\tilde{J}}_{A\beta }\cdot n_{\beta \upsilon }d\tilde{S}=\overset{}{\underset{S_{\beta \upsilon }}{\int }}{\tilde{J}}_{A\upsilon }\cdot n_{\beta \upsilon }d\tilde{S},\;\mathrm{at}\;\tilde{r}={\tilde{R}}_{P}.$$

The above equation can be tidied up by considering that $S_{\beta \upsilon }$ is a spherical surface at $\tilde{r}={\tilde{R}}_{P}$ with $d\tilde{S}={\tilde{r}}^{2}\sin\theta d\theta d\varphi$ and $n_{\beta \upsilon }=e_{r}$. Also, the radial mass flux in the β-phase is independent of the polar and azimuthal angles, while the surface averaged flux in the right hand side of Equation (8a) defines the dissolution rate from the particle surface to the υ-phase. Thus, Equation (8a) becomes

(8b) $$-{\tilde{D}}_{A\beta }{\left[\frac{d{\tilde{c}}_{A\beta }}{d\tilde{r}}\right]}_{\tilde{r}={\tilde{R}}_{P}}{\tilde{S}}_{\beta \upsilon }={\tilde{k}}_{p/\upsilon }{\tilde{S}}_{\beta \upsilon }{\tilde{c}}_{A\upsilon }\left({\tilde{R}}_{P}\right).$$

Here, the dissolution rate has been expressed in terms of the mass transfer coefficient, ${\tilde{k}}_{p/\upsilon }$, the area of the compound particle surface, ${\tilde{S}}_{\beta \upsilon }=4\pi {\tilde{R}}_{P}^{2}$, and the interfacial solute concentration at the side of the υ-phase, ${\tilde{c}}_{A\upsilon }\left({\tilde{R}}_{P}\right)$, using knowledge that will be substantiated in the following paragraphs. The value of the solute concentration at the particle surface is constant, albeit not prescribed. Substitution of the boundary condition (6d) into Equation (8b), gives

(8c) $$-{\tilde{D}}_{A\beta }{\left[\frac{d{\tilde{c}}_{A\beta }}{d\tilde{r}}\right]}_{\tilde{r}={\tilde{R}}_{P}}={\tilde{k}}_{p/\upsilon }H_{A,\upsilon /\beta }{\tilde{c}}_{A\beta }\left({\tilde{R}}_{P}\right).$$

The partition coefficient of oil at the υβ-interface, $H_{A,\upsilon /\beta }$, is approximated as the solubility ratio in the corresponding phases. By replacing Equations (6b) and (6c) with Equations (7) and (8c), respectively, and also by introducing dimless quantities, the mass transport problem defined in Equations (6a)–(6f) obtains the form

(9a) $$c_{A\beta }\left(R_{C}\right)=1,\;\mathrm{at}\;r=R_{C},$$

(9b) $$0=\frac{1}{r^{2}}\frac{d}{dr}\left(r^{2}\frac{dc_{A\beta }}{dr}\right)-h_{T}^{2}c_{A\beta },\;\mathrm{in}\;\mathrm{the}\;\upsilon \beta -\mathrm{phase},$$

(9c) $$-{\left[\frac{dc_{A\beta }}{dr}\right]}_{r=1}=\mathrm{Bi}\;c_{A\beta }\left(1\right),\;\mathrm{at}\;r=1,$$

(9d) $$c_{A\beta }\left(1\right)=c_{A\upsilon }\left(1\right),\;\mathrm{at}\;r=1,$$

(9e) $${\mathrm{Pe}}_{\upsilon }v_{\upsilon }\cdot \nabla c_{A\upsilon }=\nabla^{2}c_{A\upsilon }-{\mathrm{Da}}_{\upsilon }c_{A\upsilon },\;\mathrm{in}\;\mathrm{the}\;\upsilon -\mathrm{phase},$$

(9f) $$c_{A\upsilon }=0,\;\mathrm{at}\;r\to \infty .$$

For the non-dimensionalization, the particle radius ${\tilde{R}}_{P}$ is the reference length, the velocity $\tilde{U}$ of the approaching fluid relative to the particle is the reference velocity, the solubility of oil in the biofilm, ${\tilde{c}}_{A,\lambda /\beta }$, and in the aqueous phase, ${\tilde{c}}_{A,\lambda /\upsilon }$, is the reference concentration for the respective phase. In particular, the following dimless quantities are defined

(10a) $$r=\frac{\tilde{r}}{{\tilde{R}}_{P}};\;\nabla ={\tilde{R}}_{P}\tilde{\nabla };\;v_{\upsilon }=\frac{{\tilde{v}}_{\upsilon }}{\tilde{U}};\;c_{A\beta }=\frac{{\tilde{c}}_{A\beta }}{{\tilde{c}}_{A,\lambda /\beta }};\;c_{A\upsilon }=\frac{{\tilde{c}}_{A\upsilon }}{{\tilde{c}}_{A,\lambda /\upsilon }};$$

(10b) $${\mathrm{Pe}}_{\upsilon }=\frac{{\tilde{R}}_{P}\tilde{U}}{{\tilde{D}}_{A\upsilon }};\;{\mathrm{Da}}_{\upsilon }=\frac{{\tilde{k}}_{1\upsilon }{\tilde{R}}_{P}^{2}}{{\tilde{D}}_{A\upsilon }};\;h_{T}=\sqrt{\frac{{\tilde{k}}_{1\beta }{\tilde{R}}_{P}^{2}}{{\tilde{D}}_{A\beta }}};\;\mathrm{Bi}=\frac{{\tilde{k}}_{p/\upsilon }{\tilde{R}}_{P}}{{\tilde{D}}_{A\beta }}H_{A,\upsilon /\beta }$$

The equation set defined in Equations (9a)–(9f) can be broken down into two subproblems that can be solved independently. The external mass transport problem defined by Equations (9d)–(9f) must be solved first, in order to determine the mass transfer coefficient ${\tilde{k}}_{p/\upsilon }$ and the Biot number. As will be shown, the specific value of the solute concentration at the particle surface affects the concentration field in the υ-phase, but not the Biot number. Thereafter, the internal mass transport problem defined by Equations (9a)–(9c) must be solved in order to determine the overall dissolution rate at the surface of the oily core. 

#### 2.3.1. Advection-Dominated Transport in the Aqueous Phase without Bioreaction

In the absence of bioreaction (${\mathrm{Da}}_{\upsilon }=0$), the external mass transport problem for the unbounded aqueous domain (Ω_υ_) obtains the form

(11a) $${\mathrm{Pe}}_{\upsilon }v_{\upsilon }\cdot \nabla c_{A\upsilon }=\nabla^{2}c_{A\upsilon },$$

(11b) $$c_{A\upsilon }\left(1,\theta \right)=c_{A\beta }\left(1\right),$$

(11c) $$c_{A\upsilon }\left(\infty ,\theta \right)=0,$$

and can be solved analytically in the limits of very low (${\mathrm{Pe}}_{\upsilon }\ll 1$) or high Péclet number (${\mathrm{Pe}}_{\upsilon }\gg 1$) [37,38]. Here, the high-Péclet regime is of primary interest and, thus, the derivation of the pertinent analytical solution is outlined. In spherical coordinates, for an axisymmetric concentration field (i.e., independent of the azimuthal angle), Equation (11a) obtains the detailed form

(12) $$v_{\upsilon ,r}\frac{\partial c_{A\upsilon }}{\partial r}+\frac{v_{\upsilon ,\theta }}{r}\frac{\partial c_{A\upsilon }}{\partial \theta }=\frac{1}{{\mathrm{Pe}}_{\upsilon }}\left[\frac{\partial^{2}c_{A\upsilon }}{\partial r^{2}}+\frac{2}{r}\frac{\partial c_{A\upsilon }}{\partial r}+\frac{1}{r^{2}\sin\theta }\frac{\partial }{\partial \theta }\left(\sin\theta \frac{\partial c_{A\upsilon }}{\partial \theta }\right)\right].$$

Moreover, for creeping Newtonian flow past a rigid sphere, the velocity components are [39]

(13a) $$v_{\upsilon ,r}\left(r,\theta \right)=-\left(1-\frac{3}{2r}+\frac{1}{2r^{3}}\right)\cos\theta ,$$

(13b) $$v_{\upsilon ,\theta }\left(r,\theta \right)=\left(1-\frac{3}{4r}-\frac{1}{4r^{3}}\right)\sin\theta .$$

For advection-dominated mass transport, the change in the concentration from the value at the sphere surface ($c_{A\upsilon }=const.$) to the bulk value away from the sphere ($c_{A\upsilon }=0$) is expected to occur within a *thin* boundary layer around the sphere. Upon this consideration, the following (dimless) independent variable is introduced

(14) y≡r−1,

to measure the distance from the sphere surface, within the boundary layer. On the basis that the thickness of the concentration boundary layer is small as compared to the radius of the sphere, i.e., y≪1, the velocity terms can be simplified and certain diffusion terms can be neglected in Equation (12). In particular, order of magnitude analysis shows that the terms of tangential diffusion and normal diffusion due to surface curvature are much less important than the normal diffusion term. Under the boundary layer approximation, the final form of the *reduced* advection–diffusion equation is

(15) $$-\frac{3}{2}y^{2}\cos\theta \frac{\partial c_{A\upsilon }}{\partial y}+\frac{3}{2}y\sin\theta \frac{\partial c_{A\upsilon }}{\partial \theta }=\frac{1}{{\mathrm{Pe}}_{\upsilon }}\frac{\partial^{2}c_{A\upsilon }}{\partial y^{2}}.$$

A detailed derivation of the above equation and the development of an analytical solution by means of a similarity transformation is given in [22] (pp. 80–87) and [39] (pp. 414–417). The exact solution of Equation (15) can be expressed as follows

(16a) $$c_{A\upsilon }\left(y,\theta \right)=c_{A\beta }\left(1\right)\left[1-\frac{1}{C_{2}}\overset{\chi \left(y,\theta \right)}{\underset{0}{\int }}\exp\left(-\frac{1}{3}s^{3}\right)ds\right],$$

where $C_{2}$ is an integration constant given by

(16b) $$C_{2}=\overset{\infty }{\underset{0}{\int }}\exp\left(-\frac{1}{3}s^{3}\right)ds≅1.2879,$$

and χ(y,θ) is a composite variable defined as

(16c) $$\chi \left(y,\theta \right)={\mathrm{Pe}}_{\upsilon }^{1/3}f\left(\theta \right)y,$$

with

(16d) $$f\left(\theta \right)=\frac{\sin\theta }{{\left(\theta -\frac{\sin\left(2\theta \right)}{2}\right)}^{1/3}}.$$

The concentration field given in Equation (16) is used to determine the diffusive mass flux

(17) $${\tilde{J}}_{A\upsilon }=-\frac{{\tilde{D}}_{A\upsilon }{\tilde{c}}_{A,\lambda /\upsilon }}{{\tilde{R}}_{P}}\nabla c_{A\upsilon },$$

and, ultimately, the average mass transfer rate from the particle surface to the aqueous phase

(18) $${\tilde{W}}_{A,p/\upsilon }^{0}\equiv \overset{}{\underset{S_{\beta \upsilon }}{\int }}{\tilde{J}}_{A\upsilon }\cdot n_{\beta \upsilon }d\tilde{S}=-2\pi {\tilde{R}}_{P}{\tilde{D}}_{A\upsilon }{\tilde{c}}_{A,\lambda /\upsilon }\int_{0}^{\pi }{\left[\frac{\partial c_{A\upsilon }}{\partial r}\right]}_{r=1}\sin\theta d\theta .$$

The concentration derivative is calculated using the fundamental theorem of calculus, as follows

(19) $${\left[\frac{\partial c_{A\upsilon }}{\partial r}\right]}_{r=1}={\left[\frac{\partial c_{A\upsilon }}{\partial y}\right]}_{y=0}=\frac{\partial \chi \left(y,\theta \right)}{\partial y}{\left[\frac{dc_{A\upsilon }}{d\chi }\right]}_{\chi =0}=-\frac{{\mathrm{Pe}}_{\upsilon }^{1/3}f\left(\theta \right)}{C_{2}}c_{A\beta }\left(1\right),$$

and, after some operations, the final expression for the dissolution rate from the particle surface to the υ-phase, is given by the following expression

(20) $${\tilde{W}}_{A,p/\upsilon }^{0}={\tilde{k}}_{p/\upsilon }^{0}{\tilde{S}}_{\beta \upsilon }{\tilde{c}}_{A\upsilon }\left({\tilde{R}}_{P}\right),$$

where ${\tilde{c}}_{A\upsilon }\left({\tilde{R}}_{P}\right)={\tilde{c}}_{A,\lambda /\upsilon }c_{A\beta }\left(1\right)=H_{A,\upsilon /\beta }{\tilde{c}}_{A\beta }\left({\tilde{R}}_{P}\right)$, and

(21a) $${\tilde{k}}_{p/\upsilon }^{0}=\frac{{\tilde{D}}_{A\upsilon }}{2{\tilde{R}}_{P}}\frac{I_{\theta }}{C_{2}}{\mathrm{Pe}}_{\upsilon }^{1/3},$$

(21b) $$I_{\theta }=\int_{0}^{\pi }f\left(\theta \right)\sin\theta d\theta ≅1.6087.$$

Here, ${\tilde{k}}_{p/\upsilon }^{0}$ is the mass transfer coefficient and the “0” superscript denotes the absence of bioreaction in the bulk aqueous phase. At this point, it is very useful to introduce the *Sherwood* number which is defined as follows

(22a) $${\mathrm{Sh}}_{p/\upsilon }^{0}\equiv \frac{{\tilde{k}}_{p/\upsilon }^{0}\left(2{\tilde{R}}_{P}\right)}{{\tilde{D}}_{A\upsilon }}=1.249\;{\mathrm{Pe}}_{\upsilon }^{1/3},$$

and represents a dimless mass transfer coefficient. The above correlation underestimates the Sherwood number for about 10% for ${\mathrm{Pe}}_{\upsilon }>100$ [37] and, as expected, provides a wrong asymptotic value for ${\mathrm{Pe}}_{\upsilon }\to 0$. By simply adding the value of the Sherwood number that corresponds to diffusion-only (i.e., Sh=2 for Pe=0), the following improved correlation is obtained

(22b) $${\mathrm{Sh}}_{p/\upsilon }^{0}\equiv \frac{{\tilde{k}}_{p/\upsilon }^{0}\left(2{\tilde{R}}_{P}\right)}{{\tilde{D}}_{A\upsilon }}=2+1.249\;{\mathrm{Pe}}_{\upsilon }^{1/3}.$$

Levich suggested the above superposition based on the rationale that the resistances to mass transfer by diffusion and advection act in parallel [22]. The estimates of Equation (22b) agree with numerical data within approximately 7% for the entire range of Pe. At this point, two remarks are in order. First, the mass transfer coefficient that appears in the Biot number does not depend on the, yet unknown, interfacial concentration of the solute. Second, the definition given in Equation (18) and the final expression given in Equation (20) for the surface averaged mass transfer rate were introduced earlier in the derivation of the modified boundary condition given in Equation (8). 

#### 2.3.2. Advection-Dominated Transport and Homogeneous Bioreaction in the Aqueous Phase

Following the analysis presented previously for advection dominated mass transport under the boundary layer theory approximation, the reduced form of the advection–diffusion–reaction equation given in Equation (9e) is

(23) $$-\frac{3}{2}y^{2}\cos\theta \frac{\partial c_{A\upsilon }}{\partial y}+\frac{3}{2}y\sin\theta \frac{\partial c_{A\upsilon }}{\partial \theta }=\frac{1}{{\mathrm{Pe}}_{\upsilon }}\frac{\partial^{2}c_{A\upsilon }}{\partial y^{2}}-\frac{{\mathrm{Da}}_{\upsilon }}{{\mathrm{Pe}}_{\upsilon }}c_{A\upsilon },$$

with the same boundary conditions as given in Equations (11b)–(11c). To the best of our knowledge, an analytical solution is not available for the above partial differential equation. Approximate solutions have been developed using the empirical Ө-expansion method of Yuge, perturbation analysis, and numerical methods [37,40,41,42,43,44,45]. For engineering applications, the following simple correlation has been proposed for the Sherwood number [42,45]

(24) $${\mathrm{Sh}}_{p/\upsilon }\equiv \frac{{\tilde{k}}_{p/\upsilon }\left(2{\tilde{R}}_{P}\right)}{{\tilde{D}}_{A\upsilon }}=\frac{\mathrm{Ha}}{\tanh\mathrm{Ha}}{\mathrm{Sh}}_{p/\upsilon }^{0},$$

with

(25) $$\mathrm{Ha}=\frac{2\sqrt{{\mathrm{Da}}_{\upsilon }}}{{\mathrm{Sh}}_{p/\upsilon }^{0}},$$

where Ha is the Hatta modulus and ${\mathrm{Sh}}_{p/\upsilon }^{0}$ is the Sherwood number given by Equation (22) for the case of no bioreaction in the aqueous phase. The heuristic correlation given in Equation (24) is based on the film theory approximation and has been shown to provide an acceptable fit to more accurate numerical data. It is used here to provide estimates for the dissolution rate from the particle surface to the υ-phase, through the following relation

(26) $${\tilde{W}}_{A,p/\upsilon }={\tilde{k}}_{p/\upsilon }{\tilde{S}}_{\beta \upsilon }{\tilde{c}}_{A\upsilon }\left({\tilde{R}}_{P}\right).$$

#### 2.3.3. Diffusion and Reaction in the Biofilm Phase

The solution of the internal mass transfer problem

(27a) $$0=\frac{1}{r^{2}}\frac{d}{dr}\left(r^{2}\frac{dc_{A\beta }}{dr}\right)-h_{T}^{2}c_{A\beta },\;\mathrm{in}\;\mathrm{the}\;\beta -\mathrm{phase},$$

(27b) $$c_{A\beta }\left(R_{C}\right)=1,$$

(27c) $$-{\left[\frac{dc_{A\beta }}{dr}\right]}_{r=1}=\mathrm{Bi}\;c_{A\beta }\left(1\right),$$

is expressed as follows

(28) $$c_{A\beta }\left(r\right)=\frac{C_{3}}{r}\cosh\left(h_{T}r\right)+\frac{C_{4}}{r}\sinh\left(h_{T}r\right),$$

where $h_{T}$ is the Thiele modulus for homogeneous reaction in a spherical shell, and the integration constants are given by

(29a) $$C_{3}=\frac{\left(1-\delta_{\beta }\right)\cosh\left(h_{T}\right)\left[h_{T}+\left(\mathrm{Bi}-1\right)\tanh\left(h_{T}\right)\right]}{h_{T}\cosh\left(h_{T}\delta_{\beta }\right)+\left(\mathrm{Bi}-1\right)\sinh\left(h_{T}\delta_{\beta }\right)},$$

(29b) $$C_{4}=-\frac{\left(1-\delta_{\beta }\right)\cosh\left(h_{T}\right)\left[h_{T}\tanh\left(h_{T}\right)+\mathrm{Bi}-1\right]}{h_{T}\cosh\left(h_{T}\delta_{\beta }\right)+\left(\mathrm{Bi}-1\right)\sinh\left(h_{T}\delta_{\beta }\right)}.$$

The concentration at the particle surface (υβ-interface) is now given by the expression

(30) $$c_{A\beta }\left(1\right)=\frac{h_{T}\left(1-\delta_{\beta }\right)\sec h\left(h_{T}\delta_{\beta }\right)}{h_{T}+\left(\mathrm{Bi}-1\right)\tanh\left(h_{T}\delta_{\beta }\right)},$$

and the concentration derivative at the core surface (λβ-interface) is

(31) $${\left[\frac{dc_{A\beta }}{dr}\right]}_{r=R_{C}}=-h_{T}\left[\frac{h_{T}\tanh\left(h_{T}\delta_{\beta }\right)+\mathrm{Bi}-1}{h_{T}+\left(\mathrm{Bi}-1\right)\tanh\left(h_{T}\delta_{\beta }\right)}\right]-\frac{1}{1-\delta_{\beta }}.$$

The overall dissolution rate is

(32) $${\tilde{W}}_{A,\lambda /\beta }\equiv \overset{}{\underset{S_{\lambda \beta }}{\int }}{\tilde{J}}_{A\beta }\cdot n_{\lambda \beta }d\tilde{S}={\tilde{k}}_{\lambda /\beta }{\tilde{S}}_{\lambda \beta }{\tilde{c}}_{A,\lambda /\beta },$$

where ${\tilde{S}}_{\lambda \beta }=4\pi {\tilde{R}}_{C}^{2}$ is the area of the spherical core and the mass transfer coefficient is given by

(33) $${\tilde{k}}_{\lambda /\beta }=\frac{{\tilde{D}}_{A\beta }}{{\tilde{R}}_{P}}{\left[-\frac{dc_{A\beta }}{dr}\right]}_{r=R_{C}}.$$

Again, it is convenient to define the Sherwood number

(34) $${\mathrm{Sh}}_{\lambda /\beta }\equiv \frac{{\tilde{k}}_{\lambda /\beta }\left(2{\tilde{R}}_{P}\right)}{{\tilde{D}}_{A\upsilon }}=2\Lambda_{A\beta }h_{T}\left[\frac{h_{T}\tanh\left(h_{T}\delta_{\beta }\right)+\mathrm{Bi}-1}{h_{T}+\left(\mathrm{Bi}-1\right)\tanh\left(h_{T}\delta_{\beta }\right)}\right]+\frac{2\Lambda_{A\beta }}{1-\delta_{\beta }}.$$

The final quantity of interest is the volume averaged concentration of solute A in the β-phase

(35) $$\langle {\tilde{c}}_{A\beta }\rangle \equiv \frac{1}{{\tilde{V}}_{\beta }}\overset{}{\underset{\Omega_{\beta }}{\int }}{\tilde{c}}_{A\beta }dV,$$

which is later necessary in the determination of the particle size evolution. Here, ${\tilde{V}}_{\beta }={\tilde{V}}_{P}-{\tilde{V}}_{C}$ is the volume of the biofilm shell, with ${\tilde{V}}_{P}=\pi {\tilde{D}}_{P}^{3}/6$ and ${\tilde{V}}_{C}=\pi {\tilde{D}}_{C}^{3}/6$. After some algebraic operations, the final expression for the volume averaged concentration is

(36) $$\langle {\tilde{c}}_{A\beta }\rangle =\frac{4\pi {\tilde{R}}_{P}^{3}}{{\tilde{V}}_{\beta }}\frac{J_{C}{\tilde{c}}_{A,\lambda /\beta }}{h_{T}^{2}},$$

with

(37) $$J_{C}\equiv h_{T}^{2}\int_{R_{C}}^{1}r^{2}c_{A\beta }\left(r\right)dr={\left(1-\delta_{\beta }\right)}^{2}{\left[-\frac{dc_{A\beta }}{dr}\right]}_{r=R_{C}}-\mathrm{Bi}\;c_{A\beta }\left(1\right),$$

where the concentration and its derivative are given in Equations (30) and (31), respectively.

### 2.4. Evolution of the Particle Size: Analysis of the Overall Mass Balances

The knowledge of the oil dissolution rate at the oil–biofilm and biofilm–water interfaces as well as of the average oil concentration in the biofilm can be used to determine the change in the dimensions of the compound particle over time. This is achieved through the analysis of the *overall* mass balance for the λ- and β-phases. 

#### 2.4.1. Overall Mass Balance for the λ-Phase

Upon considering the λ-phase as an open system that may exchange mass with the surrounding phases, the integral form of the mass balance is

(38) $$\frac{d}{d\tilde{t}}\int_{\Omega_{\lambda }}^{}{\tilde{\rho }}_{\lambda }d\tilde{V}=\int_{\Omega_{\lambda }}^{}{\tilde{r}}_{A,\lambda }d\tilde{V}+\int_{S_{\lambda \beta }}^{}{\tilde{\rho }}_{\lambda }\left[{\tilde{v}}_{\lambda \beta }-{\tilde{v}}_{\beta }\right]\cdot n_{\lambda \beta }d\tilde{S}.$$

The term on the left hand side of the above equation represents the net accumulation of mass in the Ω_λ_-region. On the right hand side, the first term represents the change in the mass because of reaction in the Ω_λ_-region, and the second term represents the net influx of mass passing through the λβ-interface. Considering that the density of the λ-phase is constant, the accumulation term gives

(39) $$\frac{d}{d\tilde{t}}\int_{\Omega_{\lambda }}^{}{\tilde{\rho }}_{\lambda }d\tilde{V}=\pi {\tilde{D}}_{C,t}^{2}\frac{{\tilde{\rho }}_{\lambda }}{2}\frac{d{\tilde{D}}_{C,t}}{d\tilde{t}},$$

where ${\tilde{D}}_{C,t}=2{\tilde{R}}_{C,t}$ is the diameter of the oily core at the *t* instant of time. The direct interfacial uptake of oil is modeled as a surface reaction occurring uniformly over the droplet surface and the reaction term obtains the form

(40) $$\int_{\Omega_{\lambda }}^{}{\tilde{r}}_{A,\lambda }d\tilde{V}=\int_{\Omega_{\lambda }}^{}{\tilde{r}}_{A,\lambda \beta }\delta_{\lambda \beta }d\tilde{V}={\tilde{r}}_{A,\lambda \beta }{\tilde{S}}_{\lambda \beta ,t},$$

where $\delta_{\lambda \beta }$ is Dirac’s delta function concentrated on the λβ-interface, ${\tilde{S}}_{\lambda \beta ,t}=\pi {\tilde{D}}_{C,t}^{2}$ is the surface area of the oily core, and ${\tilde{r}}_{A,\lambda \beta }$ is the constant reaction rate given in Equation (5). 

The last term in Equation (38) represents the diffusive mass flux of oil across the λβ-interface. For completely immiscible phases, this term should be nil. For the problem at hand, the dissolution of the oil droplet is considered to be *sufficiently slow* (${\tilde{k}}_{\lambda /\beta }<<\tilde{U}$) so as not to have an appreciable impact on fluid dynamics but, nonetheless, this results in a non-zero diffusive flux across the droplet surface. Therefore, for this term we have

(41) $$\int_{S_{\lambda \beta }}{\tilde{\rho }}_{\lambda }\left[{\tilde{v}}_{\lambda \beta }-{\tilde{v}}_{\beta }\right]\cdot n_{\lambda \beta }d\tilde{S}=-\overset{}{\underset{S_{\lambda \beta }}{\int }}{\tilde{J}}_{A\beta }\cdot n_{\lambda \beta }d\tilde{S}=-{\tilde{W}}_{A,\lambda /\beta },$$

with the overall dissolution rate given by Equation (32). Substitution of Equations (39)–(41) into Equation (38), gives

(42) $$\frac{d{\tilde{D}}_{C,t}}{d\tilde{t}}=-\frac{2{\tilde{\mu }}_{m,\lambda \beta }{\tilde{B}}_{\lambda \beta }}{{\tilde{\rho }}_{\lambda }Y_{C/A,\lambda \beta }}-\frac{2{\mathrm{Sh}}_{\lambda /\beta }}{{\tilde{D}}_{P,t}}\frac{{\tilde{D}}_{A\upsilon }{\tilde{c}}_{A,\lambda /\beta }}{{\tilde{\rho }}_{\lambda }},$$

where the Sherwood number varies along with the changing particle dimensions over time. 

#### 2.4.2. Overall Mass Balance for the β-Phase

The diameter of the compound particle also changes as the dissolving oily core is shrinking and its evolution is determined by the overall mass balance for the β-phase. We have that

(43) $$\frac{d}{d\tilde{t}}\int_{\Omega_{\beta }}^{}{\tilde{\rho }}_{\beta }d\tilde{V}=\int_{\Omega_{\beta }}^{}{\tilde{r}}_{\beta }d\tilde{V}+\int_{S_{\beta \lambda }}^{}{\tilde{\rho }}_{\beta }\left[{\tilde{v}}_{\beta \lambda }-{\tilde{v}}_{\beta }\right]\cdot n_{\beta \lambda }d\tilde{S}+\int_{S_{\beta \upsilon }}^{}{\tilde{\rho }}_{\beta }\left[{\tilde{v}}_{\beta \upsilon }-{\tilde{v}}_{\beta }\right]\cdot n_{\beta \upsilon }d\tilde{S},$$

On the right hand side of the above equation, the second term could represent cell migration into the oily phase and the third term could represent the attachment of suspended cells to the biofilm or biofilm detachment and entrainment into the aqueous phase. However, for the problem at hand, both of these terms are considered to be nil. For the accumulation term on the left hand side, we have

(44) $$\frac{d}{d\tilde{t}}\int_{\Omega_{\beta }}^{}{\tilde{\rho }}_{\beta }d\tilde{V}=\pi {\tilde{D}}_{P,t}^{2}\frac{{\tilde{\rho }}_{\beta }}{2}\frac{d{\tilde{D}}_{P,t}}{d\tilde{t}}-\pi {\tilde{D}}_{C,t}^{2}\frac{{\tilde{\rho }}_{\beta }}{2}\frac{d{\tilde{D}}_{C,t}}{d\tilde{t}}.$$

The rate of change in the biofilm mass, which is caused by the growth of cells and the synthesis of the extracellular matrix, is considered to be proportional to the microbial cell proliferation rate, i.e., ${\tilde{r}}_{\beta }={\tilde{r}}_{C,\beta }/Y_{C/\beta }=-{\tilde{r}}_{A,\beta }Y_{\beta /A}$ with $Y_{\beta /A}=Y_{C/A}/Y_{C/\beta }$. Therefore, for the first term on the right hand side of Equation (43), we have

(45) $$\int_{\Omega_{\beta }}^{}{\tilde{r}}_{\beta }d\tilde{V}=Y_{\beta /A}\int_{\Omega_{\beta }}^{}\left(-{\tilde{r}}_{A,\beta }\right)d\tilde{V}=Y_{\beta /A}{\tilde{k}}_{1\beta }\int_{\Omega_{\beta }}^{}{\tilde{c}}_{A\beta }d\tilde{V}=Y_{\beta /A}{\tilde{k}}_{1\beta }{\tilde{V}}_{\beta ,t}\langle {\tilde{c}}_{A\beta }\rangle {}_{t},$$

where $\langle {\tilde{c}}_{A\beta }\rangle {}_{t}$ is the volume averaged concentration of oil in the β-phase at the *t* time instant, and is given by the expression in Equation (36). Substitution of Equations (44) and (45) into Equation (43), gives

(46) $$\frac{d{\tilde{D}}_{P,t}}{d\tilde{t}}=\frac{{\tilde{S}}_{\lambda \beta ,t}}{{\tilde{S}}_{\beta \upsilon ,t}}\frac{d{\tilde{D}}_{C,t}}{d\tilde{t}}+\frac{2{\tilde{k}}_{1\beta }Y_{\beta /A}}{{\tilde{\rho }}_{\beta }}\frac{{\tilde{V}}_{\beta ,t}}{{\tilde{S}}_{\beta \upsilon ,t}}\langle {\tilde{c}}_{A\beta }\rangle {}_{t},$$

and, further, substitution of Equation (36) for the average concentration, after some operations, gives

(47) $$\frac{d{\tilde{D}}_{P,t}}{d\tilde{t}}=\frac{{\tilde{S}}_{\lambda \beta ,t}}{{\tilde{S}}_{\beta \upsilon ,t}}\frac{d{\tilde{D}}_{C,t}}{d\tilde{t}}+\frac{4J_{C,t}}{{\tilde{D}}_{P,t}}\frac{{\tilde{c}}_{A,\lambda /\beta }}{{\tilde{\rho }}_{\lambda }}{\tilde{D}}_{A\upsilon }\Phi_{grt},$$

with

(48) $$\Phi_{grt}=\Lambda_{A\beta }Y_{\beta /A}\frac{{\tilde{\rho }}_{\lambda }}{{\tilde{\rho }}_{\beta }}.$$

#### 2.4.3. Compact and Dimless Forms of the Coupled ODEs

It is very convenient to express the coupled ordinary differential equations (ODEs) given in Equations (42) and (47), into the following compact form

(49a) $$\frac{d{\tilde{D}}_{C,t}}{d\tilde{t}}=-{\tilde{k}}_{srn}-{\tilde{k}}_{dis}\left(\tilde{t}\right),$$

(49b) $$\frac{d{\tilde{D}}_{P,t}}{d\tilde{t}}=\frac{{\tilde{D}}_{C,t}^{2}}{{\tilde{D}}_{P,t}^{2}}\frac{d{\tilde{D}}_{C,t}}{d\tilde{t}}+{\tilde{k}}_{grt}\left(\tilde{t}\right),$$

with

(50a) $${\tilde{k}}_{srn}=\frac{2{\tilde{\mu }}_{m,\lambda \beta }{\tilde{B}}_{\lambda \beta }}{{\tilde{\rho }}_{\lambda }Y_{C/A,\lambda \beta }},$$

(50b) $${\tilde{k}}_{dis}\left(\tilde{t}\right)=\frac{2{\mathrm{Sh}}_{\lambda /\beta }}{{\tilde{D}}_{P,t}}\frac{{\tilde{D}}_{A\upsilon }{\tilde{c}}_{A,\lambda /\beta }}{{\tilde{\rho }}_{\lambda }},$$

(50c) $${\tilde{k}}_{grt}\left(\tilde{t}\right)=\frac{4J_{C,t}}{{\tilde{D}}_{P,t}}\frac{{\tilde{c}}_{A,\lambda /\beta }}{{\tilde{\rho }}_{\lambda }}{\tilde{D}}_{A\upsilon }\Phi_{grt}.$$

Here, ${\tilde{k}}_{srn}$ is the droplet shrinking rate caused by direct interfacial uptake, ${\tilde{k}}_{dis}$ is the droplet shrinking rate caused by dissolution into the surrounding biofilm and aqueous phases, and ${\tilde{k}}_{grt}$ is the biofilm expansion rate due to growth. 

The Damköhler and Thiele numbers that appear in the expressions given in Equations (34) and (37) for the Sherwood number ${\mathrm{Sh}}_{\lambda /\beta }$ and the $J_{C,t}$ parameter, respectively, depend explicitly on the changing diameter of the compound particle. The situation might be a little bit more complex for the Péclet number in the case of a freely rising or sinking particle because the Stokes velocity also depends on the changing particle dimensions and density as follows

(51) $${\tilde{U}}_{S,t}=\frac{\tilde{g}}{18{\tilde{\mu }}_{\upsilon }}{\tilde{D}}_{P,t}^{2}\Delta {\tilde{\rho }}_{t},$$

where $\Delta {\tilde{\rho }}_{t}=\left|{\tilde{\rho }}_{\upsilon }-{\tilde{\rho }}_{P,t}\right|$ is the excess density of the compound particle as compared to the density of the surrounding aqueous phase, ${\tilde{\rho }}_{P,t}=\varphi_{\lambda ,t}{\tilde{\rho }}_{\lambda }+\left(1-\varphi_{\lambda ,t}\right){\tilde{\rho }}_{\beta }$ is the density of the compound particle, and $\varphi_{\lambda ,t}={\tilde{D}}_{C,t}^{3}/{\tilde{D}}_{P,t}^{3}$ is the volume fraction of the oily core. For a given set of parameters and initial conditions, the coupled ODEs given in Equations (49a) and (49b) can be solved numerically using, for instance, the explicit Euler or the classical Runge–Kutta method. In this work, both methods have been successfully implemented with an in-house Fortran code.

One step further, it is useful to establish a dimless form for the coupled ODEs that describe the evolution of the dimensions of the compound particle. For this purpose, a scaled characteristic diffusion time is introduced as follows

(52) $${\tilde{\tau }}_{D}=\frac{{\tilde{D}}_{P,0}^{2}}{{\tilde{D}}_{A\upsilon }}\frac{{\tilde{\rho }}_{\lambda }}{{\tilde{c}}_{A,\lambda /\beta }}.$$

Multiplication of both parts of Equations (49a) and (49b) with ${\tilde{\tau }}_{D}/{\tilde{D}}_{P,0}$ gives

(53a) $$\frac{dD_{C,t}}{d\tau }=-k_{srn}-k_{dis}\left(\tau \right),$$

(53b) $$\frac{dD_{P,t}}{d\tau }=\frac{D_{C,t}^{2}}{D_{P,t}^{2}}\frac{dD_{C,t}}{d\tau }+k_{grt}\left(\tau \right),$$

with

(54) $$k_{srn}={\tilde{k}}_{srn}\frac{{\tilde{\tau }}_{D}}{{\tilde{D}}_{P,0}},\;k_{dis}\left(\tau \right)=\frac{2{\mathrm{Sh}}_{\lambda /\beta }}{D_{P,t}},\;k_{grt}\left(\tau \right)=\frac{4J_{C,t}}{D_{P,t}}\Phi_{grt}.$$

For the above dimless ODEs, it is only required to define dimless ratios (diffusivity, solubility, density) and the initial values of the dimless moduli. Thereafter, at each time instant the Damköhler and the Thiele moduli are updated as follows

(55) $${\mathrm{Da}}_{\upsilon ,t}=D_{P,t}^{2}{\mathrm{Da}}_{\upsilon ,0},\;h_{T,t}=D_{P,t}h_{T,0}.$$

For the Péclet number, if the particle velocity is held constant we have ${\mathrm{Pe}}_{\upsilon ,t}=D_{P,t}{\mathrm{Pe}}_{\upsilon ,0}$, whereas if the particle is freely rising or sinking we have

(56) $${\mathrm{Pe}}_{\upsilon ,t}=D_{P,t}^{3}\Delta \rho_{t}{\mathrm{Pe}}_{\upsilon ,0},$$

with the dimless excess density given by $\Delta \rho_{t}=\left|1-\rho_{P,t}\right|/\left|1-\rho_{P,0}\right|$, and $\rho_{P,t}={\tilde{\rho }}_{P,t}/{\tilde{\rho }}_{\upsilon }$. Two final remarks are in order. First, by setting ${\tilde{\delta }}_{\beta }=0$, ${\tilde{D}}_{C,t}={\tilde{D}}_{P,t}$, and ${\tilde{c}}_{A,\lambda /\beta }={\tilde{c}}_{A,\lambda /\upsilon }$, the compound particle model degenerates into a single-phase shrinking particle model. Second, the radius is the preferred characteristic length in the analysis of the local mass balances because it naturally arises with the spherical coordinate system. On the other hand, the particle diameter is used in the evolution of the particle dimensions because this length is determined experimentally in particle size analyses.

## 3. Results and Discussion

The main outcome of the theoretical model developed in Section 2 is the expression for the overall dissolution rate for a given particle configuration and the coupled ordinary differential equations for the evolution of the dimensions of the compound particle. In this section, the effect of key system parameters on the dissolution rate and the particle size evolution are investigated.

### 3.1. Overall Sherwood Number

According to Equation (32), for a given particle configuration and constant oil solubility, the overall dissolution rate increases with increasing Sherwood number. As already mentioned, the Sherwood number ${\mathrm{Sh}}_{\lambda /\beta }$ represents the dimless mass transfer coefficient from the surface of the oily core to the surrounding biofilm shell and depends on the Biot number, the Thiele modulus, the thickness of the biofilm, and the solubility and diffusivity ratios. Among these parameters, the Biot number expresses the ratio of the external mass transfer rate (i.e., from the surface of the compound particle to the unbounded aqueous phase) over the characteristic intraparticle diffusion rate. The Biot number, in turn, depends on the Péclet and Damköhler numbers for the aqueous phase as well as on the solubility and diffusivity ratios. 

**Figure 2:**
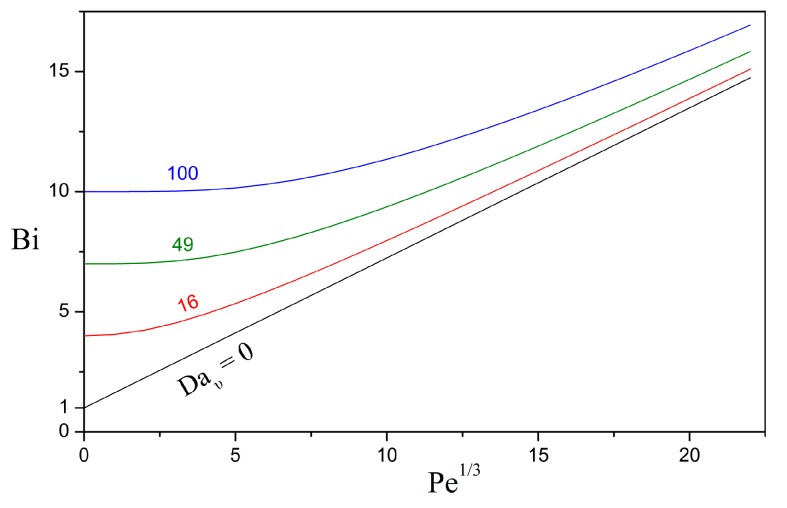
Impact of the Péclet and Damköhler numbers on the Biot number, for $\Lambda_{A\beta }=1$, $H_{A,\upsilon /\beta }=1$.

Figure 2 presents the dependence of the Biot number on the Péclet number for different values of the Damköhler number, keeping the other parameters constant. As expected, the Biot number increases with increasing Péclet and Damköhler numbers because the external mass transfer rate is enhanced by the contributions of advection and bulk bioreaction, respectively. For ${\mathrm{Pe}}_{\upsilon }=0$ and ${\mathrm{Da}}_{\upsilon }=0$, the solute moves away from the particle surface only by diffusion and the Biot number obtains the asymptotic value of $\mathrm{Bi}=H_{A,\upsilon /\beta }/\Lambda_{A\beta }$. For most solutes, diffusion within the biofilm is hindered by the extracellular matrix [46,47] and the diffusivity ratio is expected to be $\Lambda_{A\beta }\leq 1$. Furthermore, scarce experimental evidence suggests that the solubility of hydrophobic organic compounds might be significantly higher in the biofilm than in the aqueous phase ($H_{A,\upsilon /\beta }\leq 1$). In the limit of exiguous solubility in the aqueous phase, i.e., $H_{A,\upsilon /\beta }\ll 1$, the Biot number practically becomes nil and the dissolved oil is retained within the biofilm shell.

**Figure 3:**
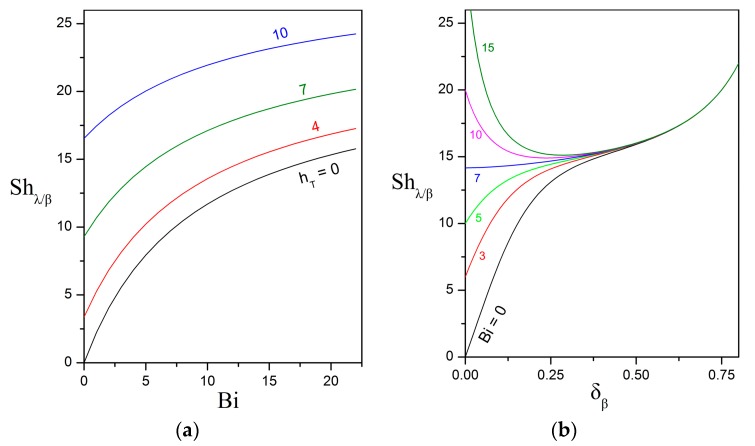
Dependence of the overall Sherwood number on: (**a**) the Biot and Thiele numbers for $\delta_{\beta }=0.1$; and (**b**) the dimless biofilm thickness and the Biot number for $h_{T}=6$. Also, $\Lambda_{A\beta }=1$.

Figure 3a shows that the overall Sherwood number increases monotonically with increasing Biot and Thiele numbers, while keeping constant the other parameters. The Thiele number is a measure of the bioreaction rate over the diffusion rate within the biofilm shell. Faster bioreaction results in a steeper concentration gradient which, in turn, drives a higher rate of oil dissolution from the surface of the oily core. Of particular interest is the case of the vanishingly small Biot number which translates into the dissolved oil remaining trapped within the biofilm until complete biodegradation is achieved. Such a function would be of great practical importance and could perhaps be implemented by biofilms with hydrophobic or lipophilic biopolymers in their extracellular matrix [48]. This aspect deserves to be examined experimentally.

Figure 3b presents a very interesting finding. If the Biot number is below a critical value (${\mathrm{Bi}}_{crit}\approx 7$ in this figure), then the overall Sherwood number increases monotonically as the dimless biofilm thickness increases. On the other hand, if the Biot number is above the critical value, then the Sherwood number decreases as the biofilm thickness increases from zero up to a critical value $\delta_{\beta ,crit}$ and, beyond that value, the Sherwood number re-increases with increasing biofilm thickness. In order to elucidate this behavior, a detailed examination of the expression given in Equation (34) for the overall Sherwood number is required. It has been established that

$${\mathrm{Sh}}_{\lambda /\beta }=2\Lambda_{A\beta }h_{T}\left[\frac{h_{T}\tanh\left(h_{T}\delta_{\beta }\right)+\mathrm{Bi}-1}{h_{T}+\left(\mathrm{Bi}-1\right)\tanh\left(h_{T}\delta_{\beta }\right)}\right]+\frac{2\Lambda_{A\beta }}{1-\delta_{\beta }}.$$

The first contribution contains the intertwined effects of transport and bioreaction in the biofilm and aqueous phases. With increasing biofilm thickness, this term increases or decreases monotonically up or down to the asymptotic value of $2\Lambda_{A\beta }h_{T}$, depending on the relative importance of the internal (biofilm) and external (aqueous) resistances to mass transport. If the internal resistance to mass transport is lower than the external resistance (i.e., sufficiently low Biot and high Thiele numbers), then the first contribution increases up to the asymptotic value as the biofilm thickness increases. In the opposite case of higher internal resistance (i.e., sufficiently high Biot and low Thiele numbers), the first contribution decreases down to the asymptotic value with increasing biofilm thickness. 

The second term in the formula for the overall Sherwood represents the effect of curvature on the concentration gradient that is evaluated at the core surface and increases monotonically with increasing biofilm thickness. In particular, each point on the oily core surface is projected to a surface element of finite area on the outer surface of the compound particle. As the distance between the two concentric spherical surfaces increases, the degree of geometric expansion also increases and causes a dilution in the solute concentration at the outer surface which, in turn, results in a higher concentration gradient. This geometric effect does not exist for a flat surface configuration. The interplay between the two contributions produces the pattern shown in Figure 3b.

The critical Biot number, above which the biofilm shell acts as a *diffusive barrier* and hinders the transport of oil compounds from the surface of the core to the surrounding aqueous phase, can be determined by the following condition

(57) $${\left[\frac{{\mathrm{dSh}}_{\lambda /\beta }}{d\delta_{\beta }}\right]}_{\delta_{\beta }=0}=0,$$

With reference to Figure 3b, the above condition states that the constant Biot curve for the critical Biot value is normal to the ${\mathrm{Sh}}_{\lambda /\beta }$ axis with abscissa $\delta_{\beta }=0$. The first derivative of the Sherwood number with respect to the dimless biofilm thickness is determined from Equation (34) and, after some operations, obtains the following form

(58) $$\frac{{\mathrm{dSh}}_{\lambda /\beta }}{d\delta_{\beta }}=\frac{2\Lambda_{A\beta }h_{T}^{2}\left[h_{T}^{2}-{\left(\mathrm{Bi}-1\right)}^{2}\right]}{{\left[h_{T}\cos h\left(h_{T}\delta_{\beta }\right)+\left(\mathrm{Bi}-1\right)\sinh\left(h_{T}\delta_{\beta }\right)\right]}^{2}}+\frac{2\Lambda_{A\beta }}{{\left(1-\delta_{\beta }\right)}^{2}}.$$

Substitution of the above expression in Equation (57), gives the following expression for the critical Biot

(59) $${\mathrm{Bi}}_{crit}=1+\sqrt{h_{T}^{2}+1}.$$

The critical biofilm thickness, below which the biofilm hinders mass transport, corresponds to the abscissa of the minimum in the constant Biot curves (for $\mathrm{Bi}>{\mathrm{Bi}}_{crit}$). Therefore, for given Biot and Thiele numbers, it can be determined as the root of the nonlinear algebraic equation that is obtained by setting the first derivative given in Equation (58) equal to zero. Another straightforward way to obtain an estimate for the critical biofilm thickness is to consider that at this value the first contribution in the Sherwood expression is *sufficiently close* to the asymptotic value of $2\Lambda_{A\beta }h_{T}$. Therefore, by demanding that $\tanh\left(h_{T}\delta_{\beta ,crit}\right)=0.99$, the following simple estimate

(60) $$\delta_{\beta ,crit}\approx \frac{2.65}{h_{T}},$$

is obtained. The critical biofilm thickness depends also on the Biot number but, as can be seen in Figure 3b, the dependence is weak and, thus, the above estimate is sufficient for practical purposes.

### 3.2. Relative Importance of the Bioreaction and Dissolution Processes

Part of the oil that dissolves at the oil–biofilm interface is biodegraded within the biofilm and the rest is released into the water column; where it might, or might not, be further degraded by suspended microbes. A key issue concerns the bioreactive effectiveness of the biofilm shell. The amount of dissolved oil that ends up either biodegraded or released can be determined by the overall mass balances (Section 2.4). 

**Figure 4:**
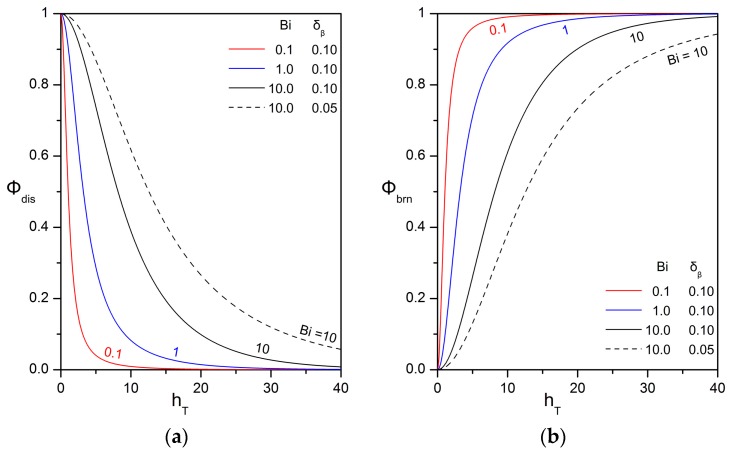
Impact of the Thiele and Biot numbers on: (**a**) the mass fraction of dissolved oil that is released into the aqueous phase; (**b**) the mass fraction of dissolved oil that is biodegraded within the biofilm. The values of the other parameters are: $\Lambda_{A\beta }=1$; $H_{A,\upsilon /\beta }=1$.

The overall dissolution rate ${\tilde{W}}_{A,\lambda /\beta }$ of oil at the surface of the oily core is given in Equation (32). The oil dissolution rate from the particle surface to the surrounding aqueous phase is given in Equation (26) and can be expressed in the following equivalent form

(61) $${\tilde{W}}_{A,p/\upsilon }={\tilde{k}}_{p/\upsilon }{\tilde{S}}_{\beta \upsilon }c_{A\beta }\left(1\right)H_{A,\upsilon /\beta }{\tilde{c}}_{A,\lambda /\beta },$$

using the relation ${\tilde{c}}_{A\upsilon }\left({\tilde{R}}_{P}\right)=c_{A\beta }\left(1\right){\tilde{c}}_{A,\lambda /\upsilon }=c_{A\beta }\left(1\right)H_{A,\upsilon /\beta }{\tilde{c}}_{A,\lambda /\beta }$, with the dimless concentration $c_{A\beta }\left(1\right)$ given in Equation (30). The rate of oil bioreaction within the biofilm can be expressed as

(62) $${\tilde{W}}_{A,\beta }\equiv \overset{}{\underset{V_{\beta }}{\int }}{\tilde{r}}_{A\beta }d\tilde{V}={\tilde{k}}_{1\beta }{\tilde{V}}_{\beta }\langle {\tilde{c}}_{A\beta }\rangle ,$$

with the average concentration of oil in the biofilm shell given by Equation (36). The mass fractions of biodegraded oil in the biofilm and released oil in the water column are defined as

(63a) $$\Phi_{brn}\equiv \frac{{\tilde{W}}_{A,\beta }}{{\tilde{W}}_{A,\lambda /\beta }}=\frac{2\Lambda_{A\beta }J_{C}}{{\left(1-\delta_{\beta }\right)}^{2}{\mathrm{Sh}}_{\lambda /\beta }},$$

(63b) $$\Phi_{dis}\equiv \frac{{\tilde{W}}_{A,p/\upsilon }}{{\tilde{W}}_{A,\lambda /\beta }}=\frac{2\Lambda_{A\beta }\mathrm{Bi}\;c_{A\beta }\left(1\right)}{{\left(1-\delta_{\beta }\right)}^{2}{\mathrm{Sh}}_{\lambda /\beta }},$$

respectively, with $\Phi_{brn}+\Phi_{dis}=1$. Figure 4 presents the effect of the Thiele modulus on the biodegraded and released oil fractions for different values of the Biot number and the thickness of the biofilm shell. It is observed that the mass fraction of biodegraded oil increases with increasing Thiele modulus, decreasing Biot number, and increasing biofilm thickness. As a consequence, biofilms composed of fast oil-degrading microbes and lipophilic extracellular matrix would be ideal for retaining and biodegrading oil compounds in practical applications.

### 3.3. Impact of the Péclet and Thiele Numbers on the Particle Size Evolution

**Figure 5:**
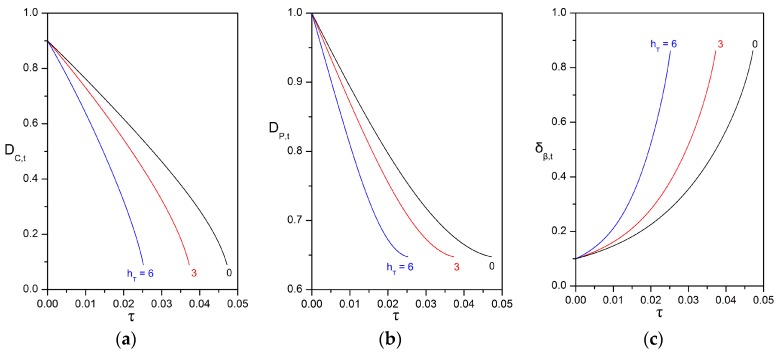
Impact of the initial Thiele modulus on the evolution of: (**a**) the dimless diameter of the oily core; (**b**) the dimless diameter of the compound particle; (**c**) the dimless thickness of the biofilm shell. The values of the other parameters are: $\delta_{\beta ,0}=0.1$; ${\mathrm{Pe}}_{\upsilon ,0}=100$; ${\mathrm{Da}}_{\upsilon }=0$; $\Lambda_{A\beta }=1$; $H_{A,\upsilon /\beta }=1$; $Y_{\beta /A}=0$; $k_{srn}=0$; velocity mode = “const. U”.

The evolution of the dimensions of the compound particle is determined by all the factors that affect the dissolution of oil into the surrounding phases, the direct uptake of oil at the surface of the oily core, and the volumetric growth of the biofilm phase. First, we examine the effects of the bioreaction in the biofilm (expressed by the Thiele number) and of the particle velocity (expressed by the Péclet number), while considering that the rates of direct uptake and biofilm growth are nil. It is very convenient to use the dimless form of the coupled ODEs given in Equations (53a) and (53b), as it is only required to define certain dimless quantities without specifying the values of solubilities, kinetic and other system parameters. Figure 5 presents the strong effect of the initial Thiele number on the evolution of the particle dimensions, while keeping all other parameters constant. As expected, higher Thiele numbers result in higher shrinking rates and faster consumption of the oily core. An interesting feature is that the temporal change in the dimensions of the particle is non-linear. For a given Thiele number, the diameter of the oily core decreases with an increasing shrinking rate (Figure 5a, concave function), whereas the diameter of the compound particle decreases with a decreasing shrinking rate (Figure 5b, convex function) until reaching an asymptotic value that corresponds to a residual particle that contains only biofilm. 

**Figure 6:**
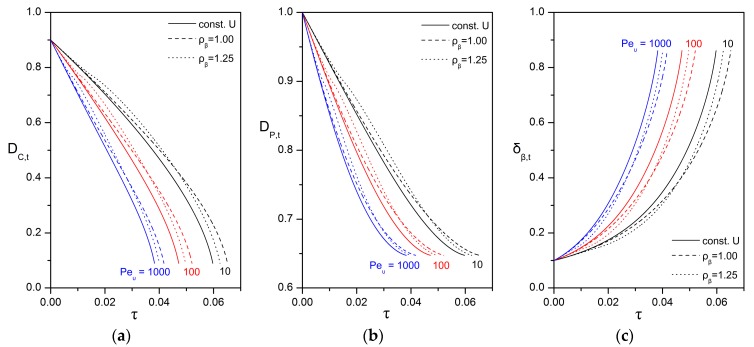
Impact of the velocity mode and the initial Péclet number on the evolution of: (**a**) the dimless diameter of the oily core; (**b**) the dimless diameter of the compound particle; (**c**) the dimless thickness of the biofilm shell. For each Péclet number, the continuous line corresponds to a compound particle that moves with constant velocity. For the free rise/fall mode: $\rho_{\beta }=1.00$ (dashed line); $\rho_{\beta }=1.25$ (dotted line). The values of the other parameters are: $\delta_{\beta ,0}=0.1$; $h_{T}=0$; ${\mathrm{Da}}_{\upsilon }=0$; $\Lambda_{A\beta }=1$; $H_{A,\upsilon /\beta }=1$; $k_{srn}=0$; $Y_{\beta /A}=0$; $\rho_{\lambda }=0.85$.

**Figure 7:**
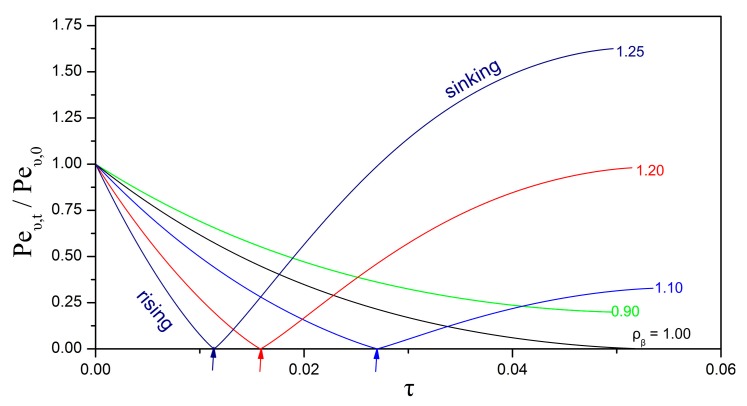
Impact of the biofilm density on the evolution of the Péclet number for a shrinking compound particle that freely rises/falls in a water column. The compound particle becomes neutrally buoyant at the time instants indicated by the arrows. The parameter values are: ${\mathrm{Pe}}_{\upsilon ,0}=100$; $\delta_{\beta ,0}=0.1$; $h_{T}=0$; ${\mathrm{Da}}_{\upsilon }=0$; $\Lambda_{A\beta }=1$; $H_{A,\upsilon /\beta }=1$; $k_{srn}=0$; $Y_{\beta /A}=0$; $\rho_{\lambda }=0.85$.

Figure 6 presents the effect of the initial Péclet number on the evolution of the particle dimensions, for the case of an inert biofilm shell (non-reactive, non-growing) and while keeping all other parameters constant. Upon the assumption that the diffusion coefficient and the initial particle size are held constant, the different Péclet numbers are associated with different characteristic velocities. Two cases are considered for the mode of change in the characteristic velocity as the particle shrinks. In the “free rise/fall” mode, the particle is considered to rise or fall in the water column under the action of gravity with the velocity given by Stokes’ formula in Equation (51) and the Péclet number updated according to Equation (56). In the “const. U” mode, the particle is considered to be carried by the aqueous stream at constant velocity. First of all, as expected, a higher Péclet number leads to faster dissolution and shrinkage of the oily core. Furthermore, for a given initial Péclet number, the velocity mode appears to have an appreciable effect on the shrinking rate. In particular, the “free rise/fall” mode exhibits an interesting dependence on the biofilm density (Figure 7). Typically, the density of the oily phase is lower than that of the surrounding aqueous phase. Therefore, oil droplets without a biofilm shell tend to rise when released in a water column. The situation is different for compound droplets because the biofilm density shows significant variability as it depends on the content and type of cells and extracellular polymers. If the biofilm density is lower than or similar to the density of the aqueous phase, then the compound particle rises with decreasing velocity and Péclet number as the oily core is consumed over time (cases of $\rho_{\beta }\leq 1$ in Figure 7). On the other hand, if the biofilm density is higher than the density of the aqueous phase, then the compound particle rises until it becomes neutrally buoyant and, thereafter, begins to sink with increasing velocity as the oily core is shrinking (cases of $\rho_{\beta }>1$ in Figure 7).

### 3.4. Impact of Biofilm Growth and Direct Uptake on the Particle Size Evolution

**Figure 8:**
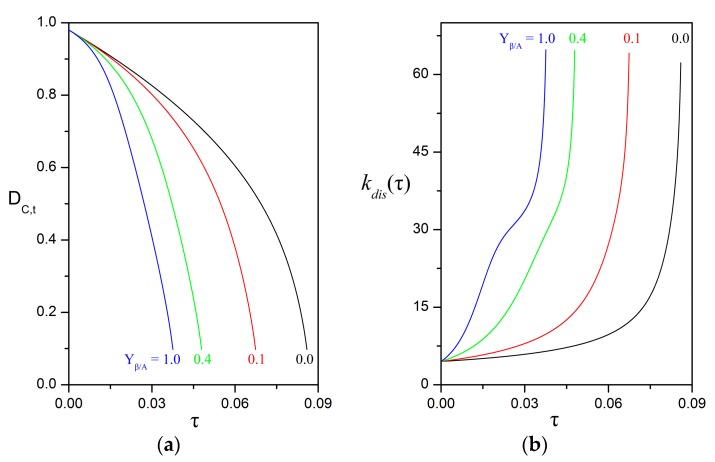
Impact of the biofilm yield coefficient $Y_{\beta /A}$ on the temporal evolution of: (**a**) the dimless diameter of the oily core; and (**b**) the dimless dissolution rate; for $H_{A,\upsilon /\beta }=0.1$; $\Lambda_{A\beta }=1$. The other parameters are: ${\mathrm{Pe}}_{\upsilon ,0}=100$; $\delta_{\beta ,0}=0.02$; $h_{T}=6$; ${\mathrm{Da}}_{\upsilon }=0$; $k_{srn}=0$; velocity mode = “const. U”.

**Figure 9:**
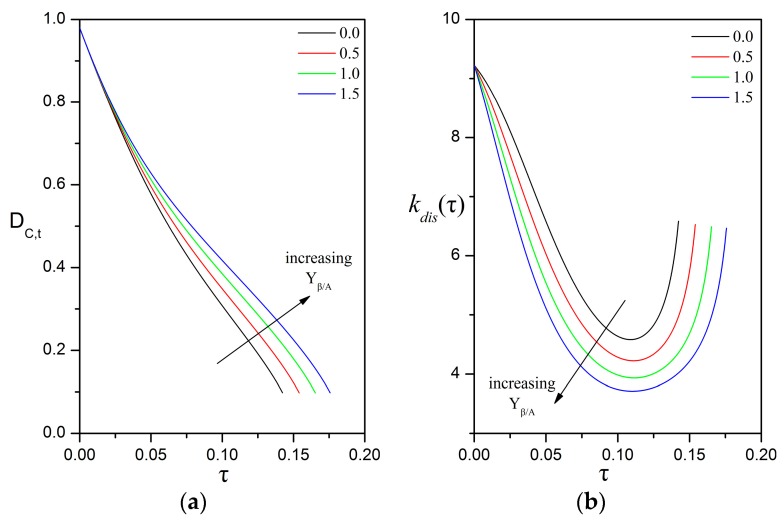
Impact of the biofilm yield coefficient $Y_{\beta /A}$ on the temporal evolution of: (**a**) the dimless diameter of the oily core; and (**b**) the dimless dissolution rate; for $H_{A,\upsilon /\beta }=1.0$; $\Lambda_{A\beta }=0.1$. The other parameters are: ${\mathrm{Pe}}_{\upsilon ,0}=100$; $\delta_{\beta ,0}=0.02$; $h_{T}=6$; ${\mathrm{Da}}_{\upsilon }=0$; $k_{srn}=0$; velocity mode = “const. U”.

The effect of a growing biofilm on the temporal evolution of the particle configuration is somewhat intricate as a thicker biofilm might not always enhance the droplet shrinking and oil biodegradation rates. If $H_{A,\upsilon /\beta }/\Lambda_{A\beta }<1.2$, that is if the oil is more soluble and mobile in the biofilm than in the aqueous phase, then the internal resistance to mass transport is also lower than the external resistance ($\mathrm{Bi}<{\mathrm{Bi}}_{crit}$) and a net increase in the amount of biofilm due to growth results in higher rates of oil dissolution and droplet shrinking (Figure 8). Under such conditions, the enhancement of the biodegradation process is more profound with thicker biofilms, i.e., for higher values of the biofilm yield coefficient $Y_{\beta /A}$. In Figure 8 and Figure 9, the value of $Y_{\beta /A}=0$ corresponds to the case where the initial amount of biofilm remains constant over time and is just redistributed around the oily core as the latter shrinks. For $Y_{\beta /A}>0$, additional volume of biofilm is produced.

On the other hand, if $H_{A,\upsilon /\beta }/\Lambda_{A\beta }>1.2$, then the internal resistance to mass transport is higher than the external resistance ($\mathrm{Bi}>{\mathrm{Bi}}_{crit}$) and a net increase in the amount of biofilm due to growth results in lower rates of oil dissolution and droplet shrinking as compared to the non-growing case (Figure 9). The peculiar trend observed in Figure 9b for the dissolution rate is attributed to the role of biofilm as a diffusive barrier, which is discussed in detail in Section 3.1. In short, the dissolution rate is defined in Equation (54) as $k_{dis}\left(\tau \right)=2Sh_{\lambda /\beta }/D_{P,t}$ and follows closely the dependence of the Sherwood number on the dimless biofilm thickness. As the oily core shrinks, the dimless biofilm thickness increases and, for $\mathrm{Bi}>{\mathrm{Bi}}_{crit}$, the Sherwood number decreases. This occurs until the biofilm exceeds the critical biofilm thickness, $\delta_{\beta }>\delta_{\beta ,crit}$, so as the enhancement caused by the curvature effect to supersede the attenuation caused by the diffusive barrier. Thereafter, the Sherwood number increases with increasing biofilm thickness (see also Figure 3b and the discussion in Section 3.1). 

**Figure 10:**
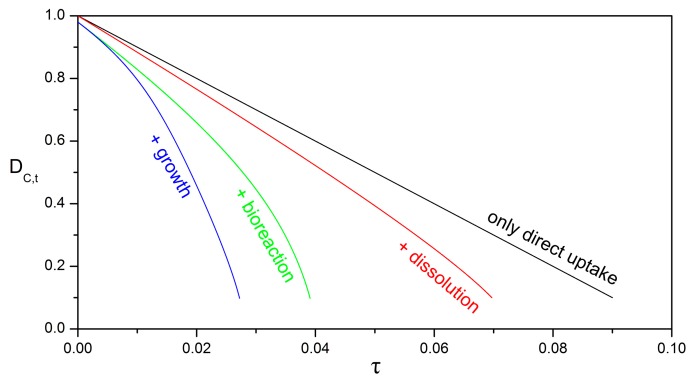
Impact of biodegradation and dissolution mechanisms on the temporal evolution of the dimless diameter of the oily core. For the scenario with only direct interfacial uptake (black line), the values of the parameters are: $k_{srn}=10$; ${\mathrm{Pe}}_{\upsilon ,0}=0$; $\delta_{\beta ,0}=0.0$; $h_{T}=0$; $Y_{\beta /A}=0$. For the scenario with added dissolution (red line), the parameters are: $k_{srn}=10$; ${\mathrm{Pe}}_{\upsilon ,0}=100$; $\delta_{\beta ,0}=0.0$; $h_{T}=0$; $Y_{\beta /A}=0$. For the scenario with added bioreaction in the biofilm (green line), the parameters are: $k_{srn}=10$; ${\mathrm{Pe}}_{\upsilon ,0}=100$; $\delta_{\beta ,0}=0.02$; $h_{T}=6$; $Y_{\beta /A}=0$. For the scenario with added biofilm growth (blue line), the parameters are: $k_{srn}=10$; ${\mathrm{Pe}}_{\upsilon ,0}=100$; $\delta_{\beta ,0}=0.02$; $h_{T}=6$; $Y_{\beta /A}=1$. In all scenarios, the other parameters are: ${\mathrm{Da}}_{\upsilon }=0$; $H_{A,\upsilon /\beta }=0.1$; $\Lambda_{A\beta }=1$; velocity mode = “const. U”.

Figure 10 illustrates the potential effect of the biodegradation and dissolution processes on the shrinking rate of the oily core. The parameter values are typical for the applications under consideration (see a detailed discussion in the next section), and have also been selected so as to exemplify that each mechanism might have a significant impact on the overall process. In real world applications, one or two or all mechanisms might act in parallel. For the direct uptake mechanism, the value of $k_{srn}=10$ is obtained in Equation (54) by setting the direct uptake rate ${\tilde{k}}_{srn}=0.2\;\mathrm{nm}/s$ (Table 1), the initial droplet diameter ${\tilde{D}}_{P,0}=100\;\mu m$, the oil density ${\tilde{\rho }}_{\lambda }=0.85\;g/{\mathrm{cm}}^{3}$, the oil diffusivity in water ${\tilde{D}}_{A\upsilon }=10^{-6}\;{\mathrm{cm}}^{2}/s$, and the oil solubility in biofilm ${\tilde{c}}_{A,\lambda /\beta }=17\;\mu g/{\mathrm{cm}}^{3}$. 

### 3.5. Implications for the Biodegradation of Crude Oil Microdroplets in the Sea

At this point, a naturally arising question concerns the values of the characteristic dimless moduli and other system parameters for real world applications. First of all, as mentioned in the introduction, the microdroplet might be rising, sinking or drifting along underwater sea currents. For light crude oil microdroplets with a diameter in the range of ${\tilde{D}}_{P,0}=10-100\;\mu m$ and a density of ${\tilde{\rho }}_{\lambda }=0.85\;g/{\mathrm{cm}}^{3}$, freely *rising* due to buoyancy through an aqueous water column with density ${\tilde{\rho }}_{\upsilon }=1.02\;g/{\mathrm{cm}}^{3}$ and dynamic viscosity ${\tilde{\mu }}_{\upsilon }=0.01\;g/\left(\mathrm{cm}\cdot s\right)$, the Stokes velocity given by Equation (51) is in the range of ${\tilde{U}}_{S,0}≅9\cdot 10^{-4}-0.09\;\mathrm{cm}/s$ and the corresponding radius-based Reynolds number is Reυ≈5·10−5−5·10−2. The density of the biofilm is expected to be similar to or larger than the density of the aqueous phase, depending on the type and volume fraction of cells and extracellular biopolymers within the biofilm. For instance, the density of marine snow particles collected from the Gulf of Mexico after the Deepwater Horizon incident exhibited great variability with values of *excess density* ranging from $0.07\;g/{\mathrm{cm}}^{3}$ to $0.36\;g/{\mathrm{cm}}^{3}$ [49]. Therefore, for compound particles with a diameter in the range of 10−100 μm and density ${\tilde{\rho }}_{P}=1.25\;g/{\mathrm{cm}}^{3}$, the *settling* Stokes velocity is also on the order of $10^{-3}-10^{-1}\;\mathrm{cm}/s$. In the case of (almost) neutrally buoyant particles, the characteristic velocity is determined by the velocity of the underwater sea current that carries the particles. The velocity of sea currents varies over several orders with a magnitude of a few mm/s for the vertical velocity [50], several cm/s for the horizontal velocity in deep sea (depth larger than 300 m) [6,50,51,52], and from tenths of cm/s up to a few m/s for the horizontal velocity near the sea surface [50,51,52]. For example, in the Gulf of Mexico, values of 2−3 mm/s have been measured for the vertical velocity [50] and an average value of 7.8 cm/s has been reported for the horizontal velocity at a depth of 1100 m [6]. Thus, for compound particles drifting along an underwater current with a velocity in the range of 0.1−10 cm/s, the Reynolds number is Reυ≈5·10−3−5.

At atmospheric conditions, the interfacial tension between crude oil and water is in the range of ${\tilde{\gamma }}_{\lambda \upsilon }=10-30\;\mathrm{dyn}/\mathrm{cm}$, depending on the detailed composition of the two phases [53,54]. An even higher tension might be expected between a compound particle and water, especially if the biofilm that covers the oily core is strongly hydrophobic. For instance, it has been recently found that microbes of the species *Bacillus subtilis* secrete a hydrophobic protein called BslA, which accumulates in the outer layer of the biofilm and results in strong repellence of aqueous drops (contact angle > 90°) [48,55]. Nonetheless, to the best of our knowledge, specific values of the interfacial tension for biofilm–water systems have not been reported yet. For a characteristic velocity in the range of $10^{-3}-10\;\mathrm{cm}/s$ and an interfacial tension of 20 dyn/cm the capillary number is $\mathrm{Ca}=5\cdot 10^{-7}-5\cdot 10^{-3}$. Furthermore, for compound particles with a diameter in the range of 10−100 μm and density of $1.25\;g/{\mathrm{cm}}^{3}$ the Bond number is $\mathrm{Bo}=3\cdot 10^{-6}-3\cdot 10^{-4}$. Finally, for a characteristic velocity in the range of $10^{-3}-10\;\mathrm{cm}/s$, and for dissolved oil components with a diffusion coefficient on the order of ${\tilde{D}}_{A\upsilon }=10^{-6}\;{\mathrm{cm}}^{2}/s$, the Péclet number is ${\mathrm{Pe}}_{\upsilon }=0.5-5\cdot 10^{4}$. In view of the above data, it is concluded that the hypotheses set out in Section 2.2 for the hydrodynamics and solute transport problems are reasonable for most of the cases considered in this work. Future extension of the CPM formulation to low-but-finite Reynolds numbers and retention of all terms in the solute mass balance is expected to improve substantially the domain of validity of the proposed model. 

With regard to the bioreaction kinetic parameters, a major issue is raised. A theoretical model, focused on a specific spatial scale, requires the input of reaction rates and parameters which precisely correspond to the scale of focus. Typically, in oil biodegradation experiments, the physical state of the oil is not taken into explicit account and an *apparent* biodegradation rate is determined in terms of an average oil concentration that lumps together all forms of oil, i.e., dissolved, micellar, and/or dispersed in droplets. This lumped concentration and its spatial and temporal derivatives differ from the concentration field that is detected by the microbes in their microenvironment [56]; in the present context, from the concentrated oil detected by flatlanders at the oil–water interface, the concentration ${\tilde{c}}_{A\upsilon }$ detected by drifters in the bulk aqueous phase, and the concentration ${\tilde{c}}_{A\beta }$ detected by biofilm formers within the biofilm. Here, the term “apparent” is used to denote a reaction rate that pertains to a representative elementary volume with dimensions much larger than the size of a single microdroplet or a single microbial cell. For systems with microscale heterogeneity, such as porous media and multiphase dispersions, the apparent reaction rate incorporates the effects of the microscale structure and transport mechanisms and is, therefore, lower than the intrinsic (transport-free) reaction rate [57]. This important issue has recently attracted the attention of researchers working on the biodegradation of crude oil [58,59,60,61,62,63,64]. For example, it has been nicely demonstrated that the apparent biodegradation rate increases with decreasing droplet size, while keeping all other system parameters constant [30,58,61]. However, existing kinetic data for apparent reaction rates of oily compounds are not consistent with the CPM formulation, and can only be used to obtain *lower bounds* for the Damköhler and Thiele numbers. 

Recent studies on the biodegradation of crude oil that was released by the Macondo well in the Gulf of Mexico after the Deepwater Horizon blowout, report that the apparent *half-life* of many biodegradable hydrocarbons is in the range of ${\tilde{T}}_{1/2}=0.1-10\;d$ (at ~ 5 °C) [7,30,62,63]. The first-order reaction constant is related to the half-life as ${\tilde{k}}_{1,\alpha }=\ln2/{\tilde{T}}_{1/2}$ and obtains values in the range of ${\tilde{k}}_{1,\alpha }\approx 0.7-7d^{-1}$. Therefore, for microdroplets of diameter ${\tilde{D}}_{P,0}=100\;\mu m$ and water diffusivity ${\tilde{D}}_{A\upsilon }=10^{-6}\;{\mathrm{cm}}^{2}/s$, the Damköhler number for the aqueous phase obtains values in the range of ${\mathrm{Da}}_{\upsilon }=2\cdot 10^{-5}-2\cdot 10^{-3}$. The diffusion coefficient of oil compounds in the interstitial space of biofilms is expected to be lower, by one or more orders of magnitude, than the water diffusivity [46,47] and, thus, the Thiele number for the biofilm phase is in the range of $h_{T}=0.0045-0.045$ (assuming $\Lambda_{A\beta }=0.1$). According to the CPM formulation, Equation (4), the first-order reaction constant depends not only on the kinetic parameters (${\tilde{\mu }}_{m,\alpha }$, ${\tilde{K}}_{S,\alpha }$, $Y_{C/A,\alpha }$), but also on the concentration ${\tilde{B}}_{\alpha }$ of active microbial cells. In natural ecosystems, the concentration of cells residing in biofilms might be several orders higher than the concentration of suspended cells. For example, after the Deepwater Horizon incident, the concentration of marine microbes in the underwater oil plume was ${\tilde{B}}_{\upsilon }=10^{4}-10^{6}\;\mathrm{cells}/{\mathrm{cm}}^{3}$ [7], whereas the cell concentration might reach values of ${\tilde{B}}_{\beta }=10^{8}-10^{10}\;\mathrm{cells}/{\mathrm{cm}}^{3}$ within marine snow and biofilms, depending on the size of individual cells and the cell volume fraction. *Vilcáez* et al. [32] developed a theoretical model for the biodegradation of droplet populations on the basis of a shrinking particle model that accounts only for the direct uptake mode. They also analyzed kinetic data from the literature and reported lumped parameters for three groups of hydrocarbons, namely alkanes, monoaromatics (BTEX), and polyaromatics (PAHs). For 100 µm-sized droplets, the data of *Vilcáez* et al. (Table 1) give values in the range of ${\mathrm{Da}}_{\upsilon }=0.0001-0.0003$ for the Damköhler number in the aqueous phase and $h_{T}=1.02-1.96$ for the Thiele number in the biofilm phase. Consequently, based on the available data, bioreaction is expected to be of considerable importance within the biofilm phase, whereas it is dominated by advection and diffusion in the aqueous phase. Nonetheless, because of the significant uncertainty with regard to the consistency between the CPM formulation and existing kinetic data for oily substrates, this discussion must be extended once data from microscale experiments become available.

## 4. Conclusions

In this paper, a compound particle model of the core-shell type is developed for the microbial degradation of solitary oil microdroplets and takes into account three fundamental biodegradation modes, namely the direct interfacial uptake at the oil surface, the bioreaction in the bulk aqueous phase, and the bioreaction in a biofilm formed around the droplet. Previous relevant models account only for the direct uptake mode. The major results of the theoretical analysis include an expression for the overall dissolution rate for a given particle configuration and two coupled ordinary differential equations for the evolution of the dimensions of the compound particle. An interesting finding is that biofilms consisting of a high concentration of fast oil-degrading microbes and lipophilic biopolymers (corresponding to a low-Biot and high-Thiele regime) are expected to be ideal for oil biodegradation applications because they retain the dissolved oil until complete degradation, instead of releasing it into the water column. The model is based on a large set of simplifying, yet justifiable, hypotheses; most of which rely on the consideration of microsized droplets with an immobilized interface by the presence of microbes and biopolymers. One of the most important hypotheses in the model is that the compound particle moves like a rigid sphere. This hypothesis negates the need to define the biofilm mechanics, which might range from a fluid-like to a solid-like behavior, depending on the composition of the biofilm and the applied stresses [65,66,67,68]. Another very important hypothesis is that the oily phase is treated as a single component, whereas crude oil and most natural or artificial oils are multi-component mixtures. Selective or faster biodegradation of certain components (e.g., alkanes) will result in concentration gradients within the oil droplet. The effects of these gradients on the droplet shrinking rate is expected to be minimal only if intra-droplet diffusion is much faster than diffusion in the surrounding biofilm phase (i.e., ${\tilde{D}}_{A\lambda }/{\tilde{D}}_{A\beta }\gg 1$). Besides the development of concentration gradients, selective biodegradation will also change the mass fraction of each oil compound within the droplet. In turn, the density of the droplet, $\rho_{\lambda }$, will also vary with time. For instance, if light alkanes are consumed faster than heavier compounds like PAHs, then the velocity of a compound particle undergoing free rise will decrease faster and the impact on the temporal evolution of the Péclet number might be appreciably stronger than that shown in Figure 7. These hypotheses will be relaxed in future work by adding more physical and mathematical complexity into the model formulation. 

## Figures and Tables

**Table 1:** Bioreaction constants (${\tilde{k}}_{1\upsilon }$; ${\tilde{k}}_{1\beta }$; ${\tilde{k}}_{srn}$) based on the kinetic parameters reported by *Vilcáez* et al. [32] for groups of hydrocarbons: ALK = alkanes; BTEX = monoaromatics (benzene, toluene, ethylbenzene, xylene); PAH = polyaromatics (naphthalene, fluorene, anthracene, etc.). For the calculation of the reaction constants, the cell concentrations are: ${\tilde{B}}_{\upsilon }=10^{6}\;\mathrm{cells}/{\mathrm{cm}}^{3}$, ${\tilde{B}}_{\beta }=10^{9}\;\mathrm{cells}/{\mathrm{cm}}^{3}$, ${\tilde{B}}_{\lambda \beta }=10^{8}\;\mathrm{cells}/{\mathrm{cm}}^{2}$.

oil	${\tilde{\mu }}_{m}\;\left[h^{-1}\right]$	${\tilde{K}}_{S}\;\left[\frac{mg}{cm^{3}}\right]$	$Y_{C/A}\;\left[\frac{cells}{mg-oil}\right]$	${\tilde{k}}_{1\upsilon }\;\left[h^{-1}\right]$	${\tilde{k}}_{1\beta }\;\left[h^{-1}\right]$	${\tilde{k}}_{srn}\;\left[\frac{\mu m}{s}\right]$
ALK	0.600	0.086	$1.25\cdot 10^{8}$	0.056	55.8	0.00314
BTEX	0.320	0.129	$1.25\cdot 10^{8}$	0.020	19.8	0.00168
PAH	0.053	0.028	$1.25\cdot 10^{8}$	0.015	15.1	0.00028

## Nomenclature

${\tilde{B}}_{\alpha }$	concentration of active cells in the αth phase, $\left[\mathrm{cells}\cdot cm^{-3}\right]$;
${\tilde{B}}_{\lambda \beta }$	interfacial concentration of active cells, $\left[\mathrm{cells}\cdot cm^{-2}\right]$;
Bi	Biot number, $Bi=H_{A,\upsilon /\beta }{\tilde{R}}_{P}{\tilde{k}}_{p/\upsilon }/{\tilde{D}}_{A\beta }=\left(H_{A,\upsilon /\beta }/\Lambda_{A\beta }\right)Sh_{p/\upsilon }/2$;
${\tilde{c}}_{A\alpha }$	mass concentration of oil in the αth phase, $\left[g\cdot cm^{-3}\right]$, dimless $c_{A\alpha }={\tilde{c}}_{A\alpha }/{\tilde{c}}_{A,\lambda /\alpha }$;
${\tilde{c}}_{A,\lambda /\alpha }$	solubility of oil in the αth phase, $\left[g\cdot cm^{-3}\right]$;
$\langle {\tilde{c}}_{A\beta }\rangle$	volume averaged concentration of oil in the β-phase, Equation (35), $\left[g\cdot cm^{-3}\right]$;
${\tilde{D}}_{A\alpha }$	diffusion coefficient of the A solute in the αth phase, $\left[cm^{2}\cdot s^{-1}\right]$;
$Da_{\alpha }$	Damköhler number in the αth phase, $Da_{\alpha }={\tilde{k}}_{1\alpha }{\tilde{R}}_{P}^{2}/{\tilde{D}}_{A\alpha }$;
${\tilde{D}}_{c,t}$	diameter of the oily core, [cm], dimless $D_{c,t}={\tilde{D}}_{c,t}/{\tilde{D}}_{P,0}$;
${\tilde{D}}_{P,t}$	diameter of the compound particle, [cm], dimless $D_{P,t}={\tilde{D}}_{P,t}/{\tilde{D}}_{P,0}$;
$\tilde{g}$	gravitational acceleration, $\left[cm\cdot s^{-2}\right]$;
$H_{A,\upsilon /\beta }$	solubility ratio, $H_{A,\upsilon /\beta }={\tilde{c}}_{A,\lambda /\upsilon }/{\tilde{c}}_{A,\lambda /\beta }$;
Ha	Hatta modulus, $Ha=2\sqrt{Da_{\upsilon }}/Sh_{p/\upsilon }^{0}$;
$h_{T}$	Thiele number in the biofilm shell, $h_{T}={\tilde{R}}_{P}\sqrt{{\tilde{k}}_{1\beta }/{\tilde{D}}_{A\beta }}=\sqrt{Da_{\beta }}$;
${\tilde{J}}_{A\alpha }$	mass flux of oil in the αth phase, $\left[g\cdot cm^{-2}\cdot s^{-1}\right]$, dimless $J_{A\alpha }={\tilde{J}}_{A\alpha }/\left({\tilde{D}}_{A\alpha }{\tilde{c}}_{A,\lambda /\alpha }/{\tilde{R}}_{P}\right)$;
${\tilde{k}}_{1\alpha }$	first-order reaction rate constant in the αth phase, Equation (4), $\left[s^{-1}\right]$;
${\tilde{k}}_{p/\upsilon }^{0}$	mass transfer coefficient for external mass transfer with $Da_{\upsilon }=0$, Equation (21), $\left[cm\cdot s^{-1}\right]$;
${\tilde{k}}_{p/\upsilon }$	mass transfer coefficient for external mass transfer, Equation (24), $\left[cm\cdot s^{-1}\right]$;
${\tilde{k}}_{\lambda /\beta }$	mass transfer coefficient for the dissolution of the oily core, Equation (33), $\left[cm\cdot s^{-1}\right]$;
${\tilde{k}}_{dis}$	droplet shrinking rate caused by dissolution, Equation (50b), $\left[cm\cdot s^{-1}\right]$, dimless Equation (54);
${\tilde{k}}_{grt}$	biofilm expansion rate due to growth, Equation (50c), $\left[cm\cdot s^{-1}\right]$, dimless Equation (54);
${\tilde{k}}_{srn}$	droplet shrinking rate caused by direct uptake, Equation (50a), $\left[cm\cdot s^{-1}\right]$, dimless Equation (54);
${\tilde{K}}_{S,\alpha }$	half-saturation constant for the A solute in the αth phase, $\left[g\cdot cm^{-3}\right]$;
$n_{\alpha \omega }$	unit normal vector on the αω-interface pointing from the α-phase to the ω-phase;
$\tilde{r}$	radial coordinate, [cm], dimless $r=\tilde{r}/{\tilde{R}}_{P}$;
${\tilde{r}}_{A,\alpha }$	oil consumption rate in the αth phase, $\left[g\cdot cm^{-3}\cdot s^{-1}\right]$;
${\tilde{r}}_{c,\alpha }$	microbial cell proliferation rate in the αth phase, $\left[\mathrm{cells}\cdot cm^{-3}\cdot s^{-1}\right]$;
${\tilde{r}}_{\beta }$	biofilm production rate, ${\tilde{r}}_{\beta }={\tilde{r}}_{c,\beta }/Y_{c/\beta }$, $\left[g\cdot cm^{-3}\cdot s^{-1}\right]$;
$Pe_{\alpha }$	Péclet number in the αth phase, $Pe_{\alpha }={\tilde{R}}_{P}\tilde{U}/{\tilde{D}}_{A\alpha }$;
${\tilde{R}}_{c}$	radius of the oily core, [cm], dimless $R_{c}={\tilde{R}}_{c}/{\tilde{R}}_{P}$;
${\tilde{R}}_{P}$	radius of the compound particle, [cm], dimless $R_{P}={\tilde{R}}_{P}/{\tilde{R}}_{P}=1$;
${\tilde{S}}_{\beta \upsilon }$	area of the compound particle surface, ${\tilde{S}}_{\beta \upsilon }=4\pi {\tilde{R}}_{P}^{2}$, $\left[cm^{2}\right]$;
${\tilde{S}}_{\lambda \beta }$	area of the oily core surface, ${\tilde{S}}_{\lambda \beta }=4\pi {\tilde{R}}_{c}^{2}$, $\left[cm^{2}\right]$;
$Sh_{p/\upsilon }^{0}$	Sherwood number for external mass transfer with $Da_{\upsilon }=0$, Equation (22);
$Sh_{p/\upsilon }$	Sherwood number for external mass transfer, Equation (24);
$Sh_{\lambda /\beta }$	overall Sherwood number for the dissolution of the oily core, Equation (34);
$\tilde{U}$	undisturbed velocity of the approaching fluid, $\left[cm\cdot s^{-1}\right]$;
${\tilde{V}}_{\beta }$	volume of the biofilm shell, ${\tilde{V}}_{\beta }=\pi \left({\tilde{D}}_{P}^{3}-{\tilde{D}}_{c}^{3}\right)/6$, $\left[cm^{3}\right]$;
${\tilde{v}}_{\upsilon }$	velocity of the aqueous fluid, $\left[cm\cdot s^{-1}\right]$, dimless $v_{\upsilon }={\tilde{v}}_{\upsilon }/\tilde{U}$;
$Y_{c/\beta }$	number of active cells per unit biofilm mass, [cells/mg-biofilm];
$Y_{c/A,\alpha }$	yield coefficient of cells in the αth phase, [cells/mg-oil];
$Y_{\beta /A}$	biofilm yield coefficient, $Y_{\beta /A}=Y_{c/A,\beta }/Y_{c/\beta }$, [mg-biofilm/mg-oil];
${\tilde{W}}_{A,p/\upsilon }$	external mass transfer rate: from the particle surface to the υ-phase, Equation (26), $\left[g\cdot s^{-1}\right]$;
${\tilde{W}}_{A,\lambda /\beta }$	overall dissolution rate at the surface of the oily core, Equation (32), $\left[g\cdot s^{-1}\right]$;
Greek letters	
${\tilde{\delta }}_{\beta }$	thickness of the biofilm shell, ${\tilde{\delta }}_{\beta }={\tilde{R}}_{P}-{\tilde{R}}_{c}$, [cm];
$\delta_{\beta }$	dimless thickness of the biofilm shell, $\delta_{\beta ,t}={\tilde{\delta }}_{\beta ,t}/{\tilde{R}}_{P,t}=1-{\tilde{R}}_{c,t}/{\tilde{R}}_{P,t}$;
$\Delta \tilde{\rho }$	excess density, $\Delta \tilde{\rho }=\left|{\tilde{\rho }}_{\upsilon }-{\tilde{\rho }}_{P}\right|$, $\left[g\cdot cm^{-3}\right]$;
${\tilde{\mu }}_{m,\alpha }$	maximum specific growth rate of active cells, $\left[s^{-1}\right]$;
${\tilde{\mu }}_{\upsilon }$	viscosity of the υ-phase, $\left[g\cdot cm^{-1}\cdot s^{-1}\right]$;
$\Lambda_{A\beta }$	diffusivity ratio, $\Lambda_{A\beta }={\tilde{D}}_{A\beta }/{\tilde{D}}_{A\upsilon }$;
${\tilde{\rho }}_{\alpha }$	density of the αth phase, $\left[g\cdot cm^{-3}\right]$, dimless $\varrho_{\alpha }={\tilde{\rho }}_{\alpha }/{\tilde{\rho }}_{\upsilon }$;
${\tilde{\tau }}_{D}$	scaled characteristic diffusion time, Equation (52), [s];
τ	dimless time,$\;\tau =\tilde{t}/{\tilde{\tau }}_{D}$;
$\Phi_{brn}$	mass fraction of oil biodegraded within the biofilm shell, Equation (63a);
$\Phi_{dis}$	mass fraction of oil released into the aqueous phase, Equation (63b);
